# Metformin and long non-coding RNAs in breast cancer

**DOI:** 10.1186/s12967-023-03909-x

**Published:** 2023-02-27

**Authors:** Morteza Gholami, Zeynab Nickhah Klashami, Pirooz Ebrahimi, Amir Ali Mahboobipour, Amir Salehi Farid, Aida Vahidi, Marziyeh Zoughi, Mojgan Asadi, Mahsa M. Amoli

**Affiliations:** 1 Metabolic Disorders Research Center, Endocrinology and Metabolism Molecular-Cellular Sciences Institute, Tehran University of Medical Sciences, Tehran, Iran; 2 Endocrinology and Metabolism Research Center, Endocrinology and Metabolism Clinical Sciences Institute, Tehran University of Medical Sciences, Tehran, Iran; 3 Department of Pharmacy, Health and Nutritional Sciences, University of Calabria, Arcavacata, Italy; 4 School of Medicine, Tehran University of Medical Sciences, Tehran, Iran; 5 Metabolomics and Genomics Research Center Endocrinology and Metabolism Molecular-Cellular Sciences Institute, Tehran University of Medical Sciences, Tehran, Iran

## Abstract

Breast cancer (BC) is the second most common cancer and cause of death in women. In recent years many studies investigated the association of long non-coding RNAs (lncRNAs), as novel genetic factors, on BC risk, survival, clinical and pathological features. Recent studies also investigated the roles of metformin treatment as the firstline treatment for type 2 diabetes (T2D) played in lncRNAs expression/regulation or BC incidence, outcome, mortality and survival, separately. This comprehensive study aimed to review lncRNAs associated with BC features and identify metformin-regulated lncRNAs and their mechanisms of action on BC or other types of cancers. Finally, metformin affects BC by regulating five BC-associated lncRNAs including GAS5, HOTAIR, MALAT1, and H19, by several molecular mechanisms have been described in this review. In addition, metformin action on other types of cancers by regulating ten lncRNAs including AC006160.1, Loc100506691, lncRNA-AF085935, SNHG7, HULC, UCA1, H19, MALAT1, AFAP1-AS1, AC026904.1 is described.

**Supplementary Information:**

The online version contains supplementary material available at 10.1186/s12967-023-03909-x.

## Introduction

**Fig. 1:**
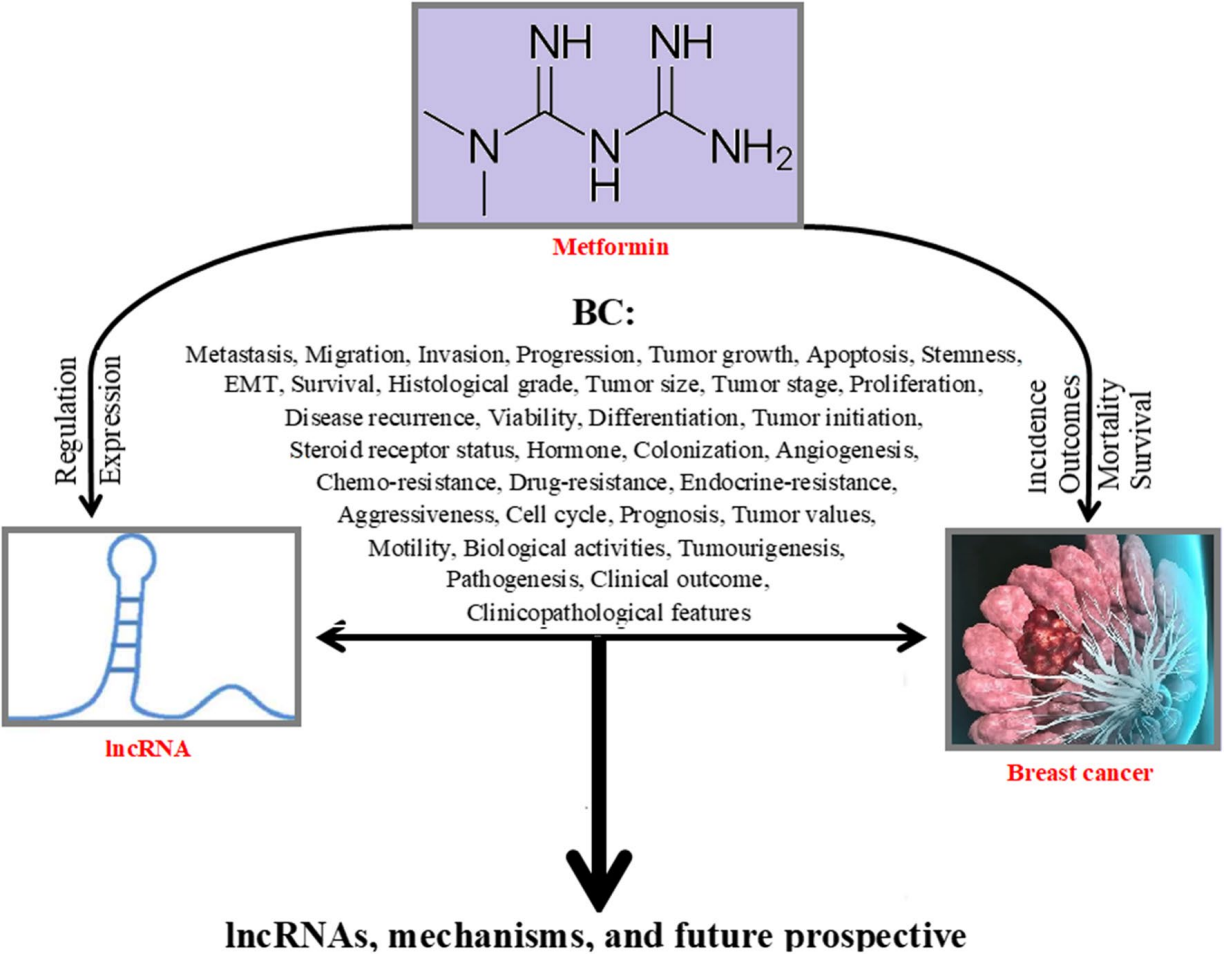
Study process

Breast cancer (BC) is one of the most important cancers in women [1]. Different types of genetics and epigenetics factors are associated with BC initiation, development, aggressiveness, and treatment. The long non-coding RNA (lncRNA) is the latest significant genetic factor affecting BC. Many studies investigated the oncogenic or tumor suppressor effects of different lncRNAs on BC features, for example, H19 is an oncogenic lncRNA that dysregulates in BC and affects different BC features including proliferation, invasion, migration, cell cycle arrest, apoptosis, metastasis, tumor values, steroid receptor status, tumor size, nodal status, disease-free survival, prognosis, stemness, mesenchymal-to-epithelial transition (MET), epithelial-to-mesenchymal transition (EMT) (Table 1 and Additional file 1: Table S1). However, while there are several studies on the role of lncRNA in BC, they investigated some limited number of BC-associated lncRNAs and there is no comprehensive review on the roles of BC-associated lncRNAs. In addition, the role of metformin (1, 1-dimethyl biguanide hydrochloride), as an anti-hyperglycemic drug, on the risk of cancers was investigated in previous studies. The protective effect of metformin on cancers and its potential use in cancer treatment or in combination with chemotherapy and radiation therapy were investigated in previous studies [2–4]. It is associated with apoptosis, cell cycle, incidence and growth of tumors [5]. Several inhibitory mechanisms of metformin are associated with BC [6, 7]. Thus, this comprehensive review examines the relationship and effects of metformin, lncRNAs, and BC on each other as shown in Fig. 1. In the first section, prevalence, mortality, types, risk factors and molecular markers of BC will be described. In the second section, history, medical uses side effects, and antitumor mechanisms of metformin will be stated. In the third section, lncRNAs history, conservation, and functions will be discussed. In the fourth section, the effects of metformin on BC incidence, mortality, survival, and outcomes will be assessed. In the fifth section, the role of lncRNAs on BC will be assessed. In the final section the association of metformin, lncRNAs, and BC is investigated and discussed.

**Table 1:** lncRNAs associated with BC

LncRNA/ID (ensemble/NCBI)	Dysregulation of lncRNA (promote/inhibit)	Oncogene/tumor suppressor	Effect on BC	References
AC026904.1 (LINC02599)/ENSG00000233858	Upregulation/promote	Oncogene	Metastasis, EMT	[111]
AFAP1-AS1/ENSG00000272620	Upregulation/promote	Oncogene	Chemoresistance, proliferation, migration, invasion	[112]
ANCR/ENSG00000226950	Upregulation/inhibit	Tumor suppressor	Invasion, metastasis, migration, EMT	[113]
DANCR/ENSG00000226950	Upregulation/promote	Oncogene	Proliferation, invasion, tumor growth	[114]
ARNILA/ENSG00000235072	Upregulation/promote	Oncogene	Progression-free survival, EMT, invasion, metastasis	[115]
ATB (LNCRNA-ATB)/Gene ID: 114004396	Upregulation/promote	Oncogene	EMT, drug resistance, anti-apoptosis, proliferation, invasion, metastasis	[116]
BC200 (BCYRN1)/ENSG00000236824	Upregulation/promote	Oncogene	Proliferation, migration, invasion, tumor size	[117]
BCAR4/ENSG00000262117	Upregulation/promote	Oncogene	Migration, invasion, metastasis	[118]
BDNF-AS/ENSG00000245573	Upregulation/promote	Oncogene	Prognostic predictor for poor survival, proliferation, endocrine resistance, progression	[119]
BLACAT1/ENSG00000281406	Upregulation/promote	Oncogene	Migration, invasion, metastasis, cell survival, proliferation	[120]
BORG	Upregulation/promote	Oncogene	Invasion, metastasis, disease recurrence	[121]
CASC2/ENSG00000177640	Upregulation/inhibit	Tumor suppressor	Proliferation, metastasis, cell cycle arrest, apoptosis, migration	[122]
CCAT1/Gene ID: 100507056	Upregulation/promote	Oncogene	Cell proliferation, migration, invasion, tumor growth, progression	[123]
CCAT2/ENSG00000280997	Upregulation/promote	Oncogene	Migration, invasion, metastasis, cancer growth, cell cycle, apoptosis, proliferation	[124]
EPIC1/ENSG00000224271	Upregulation/promote	Oncogene	Cell cycle, progression, prognosis, tumor growth	[125]
ES1 (LINC01108)/ENSG00000226673	Upregulation/promote	Oncogene	Stemness, proliferation, cell cycle progression, apoptosis, migration, EMT	[126]
EZR-AS1/ENSG00000233893	Upregulation/promote	Oncogene	Tumor growth, metastasis, prognosis, proliferation, cell cycle progression, apoptosis, migration, invasion	[127]
FEZF1-AS1/ENSG00000230316	Upregulation/promote	Oncogene	Stemness, tumorigenesis, proliferation, migration, invasion, cells growth	[128]
FGF13-AS1/ENSG00000226031	Upregulation/inhibit	Tumor suppressor	Stemness, proliferation, migration, invasion, prognosis	[129]
GACAT3/ENSG00000236289	Upregulation/promote	Oncogene	Prognosis, preoperative MRI perfusion-related diffusion reduction and elevated perfusion fraction	[130]
GAS5/ENSG00000234741	Upregulation/inhibit	Tumor suppressor	Metastasis, proliferation, drug resistance	[131]
GHET1/ENSG00000281189	Upregulation/promote	Oncogene	Proliferation, invasion, migration, apoptosis, EMT	[132]
H19/ENSG00000130600	Upregulation/promote	Oncogene	Proliferation, invasion, migration, cell cycle arrest, apoptosis, metastasis	[133]
HIF1A-AS2/Gene ID: 100750247	Upregulation/promote	Oncogene	Prognosis, migration, invasion, overall survival	[134]
HOST2 (CERNA2)/ENSG00000285972	Upregulation/promote	Oncogene	Cell motility, migration, invasion	[135]
HOTAIR/ENSG00000228630	Upregulation/promote	Oncogene	Proliferation, invasion, EMT, metastasis, drug resistance	[136]
ITGB2-AS1/ENSG00000227039	Upregulation/promote	Oncogene	Migration, invasion	[137]
LET	Upregulation/inhibit	Tumor suppressor	Proliferation, invasion, migration, apoptosis	[138]
LIMT (LINC01089)/ENSG00000212694	Downregulation/promote	Tumor suppressor	Migration, invasion, metastasis, prognosis	[139]
LINC00115/ENSG00000225880	Upregulation/promote	Oncogene	Metastasis, migration, invasion	[140]
LINC00152 (CYTOR)/ENSG00000222041	Upregulation/promote	Oncogene	Invasion, migration, tumorigenesis, colony growth, tumor growth, apoptosis	[141]
LINC00461/ENSG00000245526	Upregulation/promote	Oncogene	Invasion, migration, differentiation	[142]
LINC00511/Gene ID: 400619	Upregulation/promote	Oncogene	Proliferation, invasion, stemness, tumourigenesis	[143]
LINC00628/ENSG00000280924	Upregulation/inhibit	Tumor suppressor	Proliferation, invasion, migration, cell growth, metastasis, apoptosis	[144]
LINC00673/Gene ID: 100499467	Upregulation/promote	Oncogene	Proliferation, metastasis	[145]
LINC00899/ENSG00000231711	Upregulation/inhibit	Tumor suppressor	Proliferation, migration, invasion, progression	[146]
LINC01133/ENSG00000224259	Upregulation/inhibit	Tumor suppressor	Migration, invasion, metastasis	[147]
LINC01296 (DUXAP9)/ENSG00000225210	Upregulation/promote	Oncogene	Proliferation, prognosis, metastasis, apoptosis	[148]
LINC01787/ENSG00000231987	Upregulation/promote	Oncogene	Proliferation, migration	[149]
LINC01857/ENSG00000224137	Upregulation/promote	Oncogene	Invasion, migration	[150]
LincIN	Downregulation/inhibit	Oncogene	Migration, invasion, metastasis	[151]
Linc-ITGB1 (IATPR)/ENSG00000233387	Upregulation/promote	Oncogene	Invasion, migration, proliferation	[152]
Linc-ROR/ENSG00000258609	Upregulation/promote	Oncogene	Tumorigenesis, migration, invasion, metastasis, growth	[153]
LINP1/ENSG00000223784	Upregulation/promote	Oncogene	Apoptosis, mobility, EMT, drug resistance, migration, invasion, tumor growth	[154]
Lnc015192	Upregulation/promote	Oncogene	Migration, invasion, EMT, metastasis	[155]
Lnc01638/ENSG00000233521	Upregulation/promote	Oncogene	Proliferation, tumor growth, invasion, colonization, metastasis	[156]
Lnc-BM	Upregulation/promote	Oncogene	Metastasis	[157]
LncKLHDC7B/ENSG00000226738	Downregulation/promote	Tumor suppressor	Invasion, migration, apoptosis	[158]
LncRNA 91H	Upregulation/promote	Oncogene	Cell growth, migration, invasion, tumor growth	[159]
lncRNA HIT	Upregulation/promote	Oncogene	Migration, invasion, tumor growth, metastasis, EMT	[160]
LncRNA RP1/ENST00000420172	Upregulation/promote	Oncogene	Prognosis, proliferation, metastasis, EMT, stemness	[161]
LncRNA-ATB/Gene ID: 114004396	Upregulation/promote	Oncogene	Drug resistance, invasion-metastasis cascade	[162]
lncRNA-Hh (GAS1RR)/ENSG00000226237	Upregulation/promote	Oncogene	Tumorigenesis	[163]
Lnc-SLC4A1-1	Downregulation/inhibit	Oncogene	Apoptosis, proliferation, migration, invasion	[164]
MAGI2-AS3/ENSG00000234456	Upregulation/inhibit	Tumor suppressor	Cell growth, proliferation, cell viability, colony formation, apoptosis	[165]
MALAT1/ENSG00000251562	Upregulation/inhibit	Tumor suppressor	Migration, invasion	[166]
	Upregulation/promote	Oncogene		
			Migration, progression, proliferation, differentiation, metastasis	[167]
MAYA (MNX1-AS1)/ENSG00000243479	Upregulation/promote	Oncogene	EMT, proliferation, migration, invasion	[168]
MEG3/ENSG00000214548	Upregulation/inhibit	Tumor suppressor	Proliferation, Migration, Invasion, Apoptosis	[169]
MIR100HG/ENSG00000255248	Upregulation/promote	Oncogene	Proliferation, cell arrest in the G1	[170]
MT1JP/ENSG00000255986	Upregulation/inhibit	Tumor suppressor	Proliferation, invasion, drug sensitivity	[171]
NBAT1/ENSG00000260455	Upregulation/inhibit	Tumor suppressor	Migration, invasion, metastasis, prognosis	[172]
NEAT1/ENSG00000245532	Upregulation/promote	Oncogene	Tumor size, prognosis, proliferation, metastasis, EMT	[173]
LncRNA NEF (LNCNEF)/ENSG00000237396	Upregulation/inhibit	Tumor suppressor	Invasion, migration	[174]
NKILA/ENSG00000278709	Upregulation/inhibit	Tumor suppressor	EMT, metastasis	[175]
NLIPMT/ENSG00000278709	Upregulation/inhibit	Tumor suppressor	Proliferation, motility, progression	[176]
NNT-AS1/ENSG00000248092	Upregulation/promote	Oncogene	Migration, invasion, progression, EMT, proliferation	[177]
NORAD/ENSG00000260032	Downregulation/promote	Tumor suppressor	Migration, invasion, metastasis	[178]
P10247 (lncRNA-BCHE)/ENSG00000114200	Upregulation/promote	Oncogene	Metastasis, growth, migration, invasion	[179]
PDCD4-AS1/ENSG00000203497	Upregulation/inhibit	Tumor suppressor	Progression	[180]
PRLB (SIRLNT)/ENSG00000253802	Upregulation/promote	Oncogene	Proliferation, chemoresistance, metastasis, survival, invasion	[181]
PTENP1/ENSG00000237984	Upregulation/inhibit	Tumor suppressor	Proliferation, invasion, tumorigenesis, tumor growth, metastasis, apoptosis, chemoresistance	[182]
PVT1/ENSG00000249859	Upregulation/promote	Oncogene	Proliferation, colony formation, tumor growth	[183]
PITPNA-AS1/ENSG00000236618	Downregulation/inhibit	Oncogene	Cell viability, proliferation, migration, invasion	[184]
SPRY4-IT1/GeneID:100642175	Upregulation/promote	Oncogene	Proliferation, migration, invasion, cell cycle, apoptosis	[185]
SUMO1P3/ENSG00000235082	Downregulation/inhibit	Oncogene	Progression, survival, proliferation, migration, invasion	[186]
TFAP2A-AS1/ENSG00000229950	Upregulation/inhibit	Tumor suppressor	Invasion, migration, proliferation, cell cycle arrest, apoptosis, ability, tumor growth	[187]
TINCR/ENSG00000223573	Upregulation/promote	Oncogene	Migration, invasion, tumor growth, proliferation, apoptosis	[188]
TUG1/ENSG00000253352	Downregulation/promote	Tumor suppressor	Apoptosis, proliferation, metastasis, invasion	[189]
	Downregulation/inhibit	Oncogene	Proliferation, metastasis, tumor size, TNM staging, migration, invasion, apoptosis	[190]
TUNAR/ENSG00000250366	Upregulation/promote	Oncogene	Stemness, motility, invasion, EMT	[191]
UCA1/ENSG00000214049	Upregulation/promote	Oncogene	Apoptosis, drug resistance	[192]
XIST/ENSG00000229807	Downregulation/promote	Tumor suppressor	Cell growth, migration, invasion	[193]
LINC02095(ROCR)/ENSG00000228639	Upregulation/promote	Oncogene	Proliferation	[194]
WT1-AS/ENSG00000183242	Upregulation/inhibit	Tumor suppressor	Clinical stages, migration, invasion	[195]
LINC00096(TP53TG1)/ENSG00000182165	Upregulation/promote	Oncogene	Proliferation, invasion, metastasis	[196]
HEIH/ENSG00000278970	Downregulation/inhibit	Oncogene	Proliferation, apoptosis	[197]
LUCAT1/ENSG00000248323	Upregulation/promote	Oncogene	Proliferation, cell cycle progression, metastasis, apoptosis	[198]
ASRPS(LINC00908)/ENSG00000266256	Downregulation/promote	Tumor suppressor	Angiogenesis, tumor growth	[199]
HAND2-AS1/ENSG00000237125	Upregulation/inhibit	Tumor suppressor	Proliferation	[200]
LINC01096/ENSG00000246095	Downregulation/inhibit	Oncogene	Proliferation, migration, invasion; apoptosis, cell viability	[201]
PANDA(PANDAR)/ENSG00000281450	Upregulation/promote	Oncogene	Apoptosis	[202]
TP73-AS1/ENSG00000227372	Upregulation/promote	Oncogene	Proliferation, invasion, migration	[203]
CRNDE/ENSG00000245694	Upregulation/promote	Oncogene	Proliferation, migration, invasion	[204]
HCP5/ENSG00000206337	Downregulation/promote	Tumor suppressor	Drug resistance	[205]
ADAMTS9-AS2/ENSG00000241684	Downregulation/promote	Tumor suppressor	Drug resistance, apoptosis, viability	[206]
TMPO-AS1/ENSG00000257167	Upregulation/promote	Oncogene	Proliferation, viability, apoptosis, drug resistance	[207]
DSCAM-AS1/ENSG00000235123	Downregulation/inhibit	Oncogene	Proliferation, colony formation	[208]
MAFG-AS1(MILIP)/ENSG00000265688	Downregulation/inhibit	Oncogene	Proliferation, apoptosis, drug resistance	[209]
DILA1(MIR99AHG)/ENSG00000215386	Upregulation/promote	Oncogene	Drug resistance, proliferation, prognosis, tumor growth	[210]
DLX6-AS1/ENSG00000231764	Upregulation/promote	Oncogene	Apoptosis, migration, drug resistance, EMT	[211]
SNHG7/ENSG00000233016	Upregulation/promote	Oncogene	Viability, drug resistance	[212]
DCST1-AS1/ENSG00000232093	Upregulation/promote	Oncogene	Drug resistance, EMT, chemoresistance	[213]
LINC00472/ENSG00000233237	Downregulation/promote	Tumor suppressor	Growth, aggressiveness	[214]
AGAP2-AS1/ENSG00000255737	Upregulation/promote	Oncogene	Tumor growth, apoptosis, chemoresistance, drug resistance	[215]
SNHG14/ENSG00000224078	Upregulation/promote	Oncogene	Proliferation, invasion, drug resistance	[216]
MAPT-AS1/ENSG00000264589	Downregulation/inhibit	Oncogene	Drug resistance, migration, invasion, proliferation	[217]
Linc00518/ENSG00000183674	Upregulation/promote	Oncogene	Drug resistance	[218]
FTH1P3/ENSG00000213453	Downregulation/inhibit	Oncogene	Drug resistance	[219]
FGF14-AS2/ENSG00000272143	Downregulation/promote	Tumor suppressor	Progression, prognosis, tumor size, lymph node metastasis, clinical stage, overall survival	[220]
PAPAS	Upregulation/promote	Oncogene	Migration, invasion	[221]
lncMat2B	Upregulation/promote	Oncogene	Drug resistance	[222]
LOL	Downregulation/inhibit	Oncogene	Apoptosis, proliferation, drug resistance	[223]
BC032585	Downregulation/promote	Tumor suppressor	Drug resistance	[224]
NONHSAT101069	Upregulation/promote	Oncogene	Drug sensitivity, metastasis, migration, invasion	[225]
DRHC	Downregulation/promote	Tumor suppressor	Proliferation	[226]

## BC

The first evidence of BC dates back to 2500–3000 BC. There is also evidence of BC in Hippocrates' manuscripts. It is the second most common type of cancer in women in the United States [1]. The lifetime risk of developing BC is one out of every eight women [8]. About 264,000 new cases of BC in women are diagnosed in the United States every year [1]. After lung cancer, it is the second cause of death from cancers in women. The number of deaths from this cancer in women is about 42,000 each year [1], and 15% of women deaths between the age of 20 to 59 years occur due to BC [9]. Male BC is not common and makes up only one percent of all patients with BC. Although the incidence of BC is lower in men, the prognosis is worse [10].

Based on the cell origin, BC is carcinoma or sarcoma. Carcinomas arise from the epithelial components including the cells that line the lobules and ducts responsible for milk production. Sarcomas, a rarer form, arise from the stromal components including myofibroblasts and blood vessel cells [11]. Based on the criteria of pathological characteristics and aggressiveness, BC is graded as non-invasive/preinvasive (intraductal carcinoma, most common type), invasive, and metastatic. There are two ductal and lobular types of BC. The ductal form involves milk ducts. But in the lobular form, the milk-producing glands are involved. These types are also divided into invasive (or infiltrating) and in-situ forms based on their spread pattern. Unlike the in-situ form, the invasive form invades the surrounding tissues [12]. The invasive ductal carcinoma (80% of BC) includes tubular, medullary, mucinous, papillary, and cribriform carcinoma subtypes, while invasive lobular carcinoma (10–15% BC) is reported more in women with increasing age [11]. Also, there are two special invasive types of BC including triple-negative and inflammatory. In triple-negative BC (TNBC), cancerous cells do not have estrogen receptor (ER), progesterone receptor (PR), and human epidermal growth factor receptor (EGFR) which leads to poor survival outcomes [13]. TNBC common treatments are lumpectomy, mastectomy, radiation therapy, and chemotherapy. The human epidermal growth factor receptor 2 (HER2) is not present in these cells [10]. In the inflammatory form, lymphatic vessels are also blocked, which is a very aggressive form [14].

The breast completes its development in puberty and pregnancy through alterations in breast differentiation and proliferation with more ductal branching and lobuloalveolar growth. The balance between differentiation, proliferation, and apoptosis is critical for the normal development and homeostasis of breast cells. The premalignant stages change this balance and the regulatory biomarkers of the cell cycle. Change in this may lead to the accumulation of mutations in BC [15]. Uncontrolled cell proliferation in tumors is associated with lower apoptosis. Apoptosis is a marker of prognosis, the low level of apoptosis is a poor prognostic feature while high levels of apoptosis in tumors have been correlated with the absence of estrogen receptors and worse survival [15]. The apoptosis-associated proteins are involved in BC and its treatment. The Bad, Bak, Bax, and Bcl-xs are pro-apoptotic proteins and Bcl-2 and Bcl-xL are anti-apoptotic proteins. Tumor Protein 53 (P53) is associated with a poor prognosis of BC and poor response to chemotherapy. The XIAP, NIAP, cIAP1, cIAP2, and survivin proteins prevent apoptosis by directly inhibiting caspases [15].

Metastasis is associated with most cases of cancer mortality which genetically leads migration of cancerous cells to other organs by the blood and lymphatic vessels [9, 16]. It is associated with processes including MET, EMT, cancer cell migration, invasion, proliferation, stemness, angiogenesis, anoikis [16], endothelial cells, macrophages, extracellular matrix, stem cells [17] and cancer-associated fibroblasts [18]. The microvascular production increase in tumor cells and is associated with poor prognosis of BC. The hypoxia and genetic changes in the tumor cells are related to an increase in angiogenic factors [19]. The hypoxic tumor HIF-1α induces the expression of proangiogenic mediators [20]. Different factors such as Hypoxia, HIF-1α, HER2, and matrix metalloproteinase 9 (MMP-9) increase the expression of VEGF in BC, and VEGF is associated with angiogenesis in breast tumors. VEGF is involved in the initial BC tumor growth while bFGF is increased during further tumor growth. The FGF/FGFR signaling as an angiogenic driver is associated with BC therapeutic perspectives [21]. In general, different tissues are different in the angiogenic response.

Several risk factors have been identified for BC. However, more than 50% of affected people had no risk factors except for increasing age and female gender [10]. In general, these risk factors can be classified into two categories: changeable and non-changeable. The most important non-changeable risk factors are genetics, increasing age, female gender, race, taller height, first-degree family history of BC, benign BC, early menarche less than 12 years old, or late menopause older than 55 years old. The most important changeable risk factors include risk factors related to lifestyle and personal behavior, long-term hormone replacement therapy, chest wall irradiation, obesity, high-fat diet, and environmental chemicals. Although a variety of risk factors have been discovered, the exact causes of breast cancer are not yet entirely understood.

The “tumor biomarker” is a possible indicator of the onset, development, and progression of cancer. We must examine these biomarkers deeper and work to understand the underlying processes of tumor formation to improve treatment and personalized medicine [22]. The predictive and prognostic biomarkers, such as circulating tumor cells (CTCs), DNA, RNA, and miRNA, have been connected to probable clinical outcomes and treatment effectiveness of BC. Intact cells and larger molecules such as nucleic acids, genetic changes, and protein molecules are used as molecular, histological, radiographical, or physiological biomarkers in the detection of cancer [23]. Investigating molecular markers leads to the improvement of BC screening, diagnosis, and treatment processes. BC biomarkers are classified into two tumors or blood categories. Different regulatory noncoding RNAs such as miRNAs, lncRNAs, and piRNAs play non-invasive biomarkers roles in BC development, diagnosis, and prognosis. Inappropriate circulating mRNAs expression is associated with BC as a tumor marker, for example 5T4 circulating RNA may potentially be used to identify patients who can benefit from a 5T4 therapy utility of circulating RNA [24], or circulating circular RNA hsa_circ_0001785 upregulation in BC which could be a biomarker for BC diagnosis and progress [25]. The circulating cell-free miRNAs in the serum of BC patients emerged as a promising new noninvasive biomarker for the early detection of tumors and for predicting their molecular classifications. These miRNAs target mRNA that encodes proteins involved in different molecular pathways such as, proliferation, cell adhesion, and migration [26]. miRNA:mRNA interactions are associated with the invasiveness of BC [27]. miRNAs are suggested to be associated with BC therapeutic approaches [28], prognosis [29], progression, and metastasis [30]. P-element-induced wimpy testis (PIWI) interacting RNAs (piRNAs) are a novel type of non-coding RNAs, with act on both transcription and post-transcription. Upregulation of piR-021285, piRNA-823, piRNA-932, piR-016658, piR-651, piR-4987, piR-20365, piR-20485, and piR-20582 were associated with EMT, invasiveness, metastasis, lymph node metastasis, while downregulation of piR-36712, piR-016975, piR-FTH1 in BC tissue were related to EMT, chemosensitivity, and chemoresistance [31]. PiRNA-mediated epigenetic mechanism and altered DNA methylation are involved in BC tumorigenesis [32]. The function and mechanism of many piRNAs in cancers is unclear. The role of lncRNAs as one of the most important noncoding RNAs in BC will be fully discussed in “lncRNAs in BC” section.

## Antitumor mechanisms of metformin in BC

Metformin is a synthetic derivative of galegine and/or guanidine and belongs to a group of compounds called biguanides. galegine synthesize from *Gallega officinalis*, a plant that has been used for centuries in Europe to treat diabetes [33]. Metformin works by improving insulin sensitivity in patients with type 2 diabetes (T2D) due to its biguanide properties [34]. For the first time, metformin was used to treat T2D in the 1950s, but from 1995 it became widely used in the United States as a first-line treatment [35]. It is an anti-hyperglycemic drug (lowering blood sugar level) and the first line of treatment for T2D. The reduction of blood sugar level is done by inhibiting hepatic glucose production, increasing glucose absorption, and its consumption by skeletal muscles [36]. In addition, Metformin reduces insulin resistance in the surrounding tissue and suppresses gluconeogenesis in the liver.

Metformin has pleiotropic effects and is beneficial in the treatment of diabetes in various diseases, including prediabetes, and type 1 diabetes mellitus [37]. Many previous studies, demonstrate the safety and well-tolerance of metformin, along with potential nephroprotective and cardioprotective effects [38]. There are several other indications for metformin that are not FDA-approved, including the treatment of gestational diabetes, the treatment of weight gain caused by antipsychotics, the prevention of type 2 diabetes, as well as the treatment and prevention of polycystic ovary syndrome [39]. However, in some specific populations, such as patients with renal or hepatic impairment, pregnant or breastfeeding women, and pediatric or geriatric patients, metformin usage may not be commonly safe. The primary potential of lactic acidosis production made it unsafe in some circumstances [40]. It is important to understand the benefit-risk balance of metformin treatment when it comes to elderly patients who are highly likely to have stable renal impairment, congestive heart failure, and/or coronary artery disease due to the high prevalence of these conditions.

Although no serious complications of metformin have been observed, this drug has some side effects like any other drug. There may be symptoms such as dizziness, severe drowsiness, muscle pain, tiredness, chills, blue/cold skin, rapid or difficult breathing, slow heartbeat, stomach pain associated with diarrhea, and a feeling of nausea and vomiting. In most cases, lactic acidosis is caused by an overdose of medication or is the result of some contraindications. Metformin rarely causes hypoglycemia, however, if it is used in conjunction with other anti-diabetic medications, low blood sugar may develop. In rare cases, it leads to a serious allergic reaction. The product contains inactive components that may cause reactions or other problems when administered to patients.

The risk of developing cancer in diabetic patients taking metformin is reduced compared to other people, and its protective effect increases with exposure to a higher dose of metformin [2]. Metformin reduces cell proliferation, induces apoptosis, stops the cell cycle in vitro, and also reduces the incidence and growth of tumors in vivo [3]. It can be used as a sensitizing agent or in combination with chemotherapy and radiation therapy to fight cancer [4]. There are several mechanisms by which metformin inhibits the proliferation of malignant cells, such as activation of AMP-activated protein kinase (AMPK) or Mitogen-activated protein kinases (MAPK), decrease in mechanistic target of rapamycin (mTOR) signaling, increase in p27 expression, protein synthesis, EGFR, Src, and expression of Cyclins. Controlling the expression levels of proteins that are necessary for the transition between the G1 and S cell cycle, including cyclin D1, cyclin E1, and E2F transcription factor 1 [6, 7]. Metformin inhibits Cyclin-dependent kinases and causes the cell cycle to stop in the G1 phase [41]. The ability to deactivate biosynthetic mitochondrial nodes in cells with the BRCA1 gene is a potential mechanism of action of metformin for suppressing the BC formation in cell types affected by BRCA1 [42]. It was also proven that BRCA1 haploinsufficiency leads to the activation of AKT/mTOR-mediated protein synthesis driven by hyperphosphorylation of the BRCA1 substrate rich in proline through AKT activity. Another interesting function of metformin is the disruption of AKT/mTOR-signaling network in BRCA1 haploinsufficient cells [43]. Decreasing insulin and insulin-like growth factor levels inhibit signaling that involve phosphoinositide 3-kinase, AKT, and mTOR, it may be related to the inhibition of mTOR signaling by activating AMPK [39]. A major target of metformin therapy is the regulation of AMPK through an AMP-dependent signaling pathway. AMPK activation inhibits cell mitosis and proliferation [44]. Metformin exerts its anti-neoplastic effects by stimulating AMPK through up-regulation of p53–p21 and down-regulation of Cyclin D1 levels. The activation of AMPK, through inhibition of the mTOR, activity, fatty acid synthesis signaling pathways, as well as stimulating the apoptotic pathway (p53/p21) is responsible for regulating tumor cell survival and tumor growth [5]. The neoadjuvant metformin administration in BC decreased insulin receptors, phosphorylation of protein kinase B (PKB)/Akt, AMPK, and extracellular signal-regulated kinase1/2. This insulin-dependent effect of metformin is consistent with its anticancer properties [45]. Metformin has numerous beneficial properties in both normal and cancerous cells, including reduced insulin levels, inhibition of insulin/IGF signaling pathways, as well as modulation of cellular metabolism [46]. There are several roles played by insulin/IGF-1 in the regulation of glucose uptake, as well as the regulation of carcinogenesis through the upregulation of signaling pathways associated with insulin/IGF receptors [47]. The signal through the insulin receptor substrate phosphorylates (but does not activate) mTORC1. Furthermore, through growth factor receptor-bound protein 2, insulin signals are transmitted to Ras/Raf/ERK pathway which is responsible for regulating cell growth [48]. Several studies have indicated that these pathways play a significant role in the changes that occur in the metabolism of cancer cells [49]. A significant decrease in IGF-1/insulin receptor activity, Akt, extracellular signal-regulated kinase (ERK) activity, and AMPK activity is observed with metformin use without activation of AMPK by this drug [46]. It has been shown that metformin inhibits mTOR signaling in Drosophila cells when AMPK is absent. There is an alternative pathway mediated by the RAG GTPase associated with tuberous sclerosis protein (TSC1/2-mTOR) which is AMPK independent [50]. On the other hand, as a result of decreasing the levels of HER2 in breast cancer cells, metformin can inhibit breast carcinoma cell growth, and inhibition of p70S6K1, an effector of the mTOR pathway, can mediate this effect [51].

**Fig. 2:**
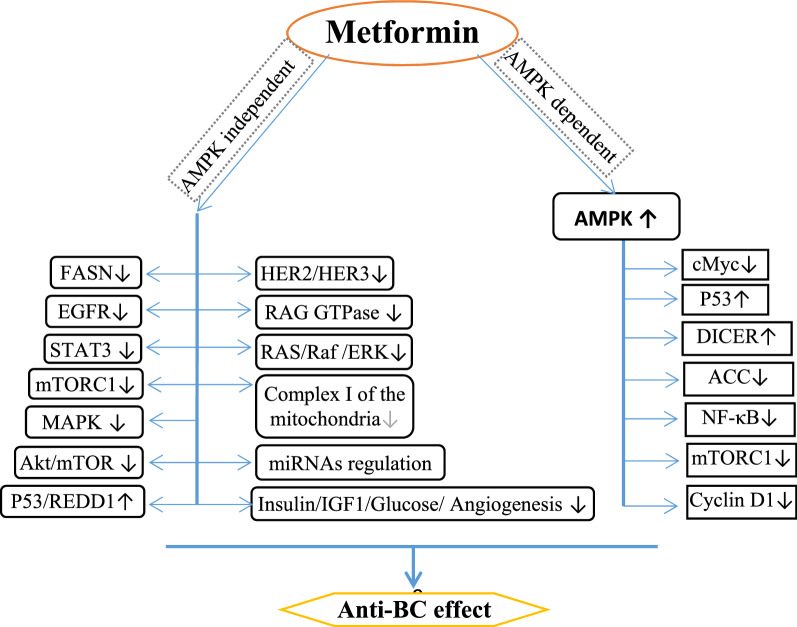
Anti-BC effects of metformin

Hyperglycemia induces oxidative stress both directly and indirectly in BC cells in part by increasing levels of insulin/IGF-1 as well as inflammatory cytokines [52]. Furthermore, the activation of nuclear factor kappa (NFκB), signal transducer activator of transcription 3 (STAT3), and the hypoxia-inducible factor 1-alpha (HIF1α) are involved [53]. Through anti-inflammatory effects in cell models, metformin inhibits the components of the NFκB pathway that are essential for the transformation of stem cells and the formation of cancer stem cells. Metformin also prevents phosphorylation of STAT3 in cancer stem cells [54, 55]. The suppression of chronic inflammatory response by metformin is related to the inhibition of TNF-α production in human monocytes and chronic inflammation provides a basis for cancer development [56]. Metformin decreases cellular-Myc (c-Myc) and increases double-stranded RNA specific endoribonuclease (DICER) in AMPK signaling [57]. Metformin leads to reprograming of lipid metabolism, as a hallmark of cancer, by increase in acetyl-CoA carboxylase (ACC) and fatty acid synthase (FASN), and miRNA regulation [58]. It also inhibits Complex I of the mitochondria and increases the AMP/ATP ratio which leads to further AMPK activation [59]. The anti-BC effects of metformin have been illustrated in Fig. 2.

Autophagy is another way to prevent the growth of malignant cells. Autophagy initiates the formation of membrane structures, including autophagosomes, by recruiting a family of autophagy-related (ATG) proteins [60]. It has been suggested that metformin may have antitumor effects in part due to its ability to increase levels of ATG3, ATG5, and LC3-II in cells treated with metformin [61]. In order to repurpose metformin for the treatment of BC, epigenetic regulators would be modulated. Previous studies showed that metformin treatment altered the abundance of RBBP4, G9a, acH3K9, and acH3K18, suggesting that histone modifiers may play an important role in metformin cancer treatment [62].

Obesity, diabetes, and hyperinsulinemia are associated with an increased risk of BC [63]. Fat distribution in the body, BMI, and weight changes are among the factors affecting BC [64]. Several factors such as adipokines, IGFs (IGF-1 as mitogens), dyslipidemia, hyperglycemia, hyperinsulinemia, and inflammatory cytokines link BC with obesity and diabetes [63]. IGF-1 gene expression increases in people with breast cancer compared to healthy people, while the circulating levels of Insulin-like growth factor binding protein 3 (IGFBP-3) will decrease [65]. The level of circulating insulin decreases in diabetes while it increases in obesity and cancers [66]. Insulin resistance is involved in obesity and diabetes and contributes to the development of BC. Metformin decreases insulin resistance, while obesity and diabetes both induce insulin resistance which leads to hyperinsulinemia. Hyperinsulinemia increases IGF-1 and decreases IGFBP1/2 (that finally cause dyslipidemia or increased bioavailable estrogen), or induce β-cells failure and hyperglycemia to finally increase BC cell growth [63]. Hyperglycemia increases the production of free radicals, damage and mutations in oncogenes and tumor suppressor genes and finally proliferation of cancer cells [67]. In addition, obesity and diabetes increase BC cell growth by upregulation of inflammatory cytokines such as IL-6, IL-1β, TNF-α [63].

Despite the fact that BC is well known to be associated with metabolic characteristics of T2D, including hyperglycemia, hyperinsulinemia, inflammation, oxidative stress, and obesity, randomized controlled trials show opposite results for metformin as an insulin sensitizer [59]. Considering that glucose is a crucial cellular metabolic substrate and insulin signaling has mutagenic effects on BC, growing and spreading BC are intimately linked to glucose metabolism [68]. The growing evidence suggests that metformin may play a preventative role in BC. Metformin counteracts insulin-stimulatory effects and leads to anti-proliferative and anti-migratory effects in primary breast cancer cells [69]. The first evidence that metformin might have antitumor properties was discovered by Evanns et al. in 2005 after they found that T2D patients who were taking metformin had a lower risk of malignancy [70].

**Fig. 3:**
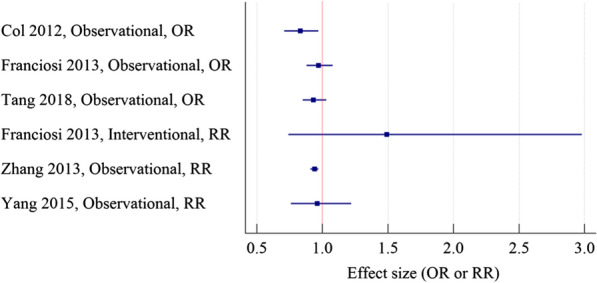
Meta-analyses on the association between metformin and BC incidence in diabetic patients. *OR* odds ratio, *RR* risk ratio

In the meta-analyses on the association between metformin use and BC incidence, the preventive effect of metformin was observed in Col 2012 and Zhang 2013 meta-analyses [71]. However, the protective effect of metformin on BC prevention was not confirmed in subsequent meta-analyses [72–75]. The results are shown in Fig. 3.

**Fig. 4:**
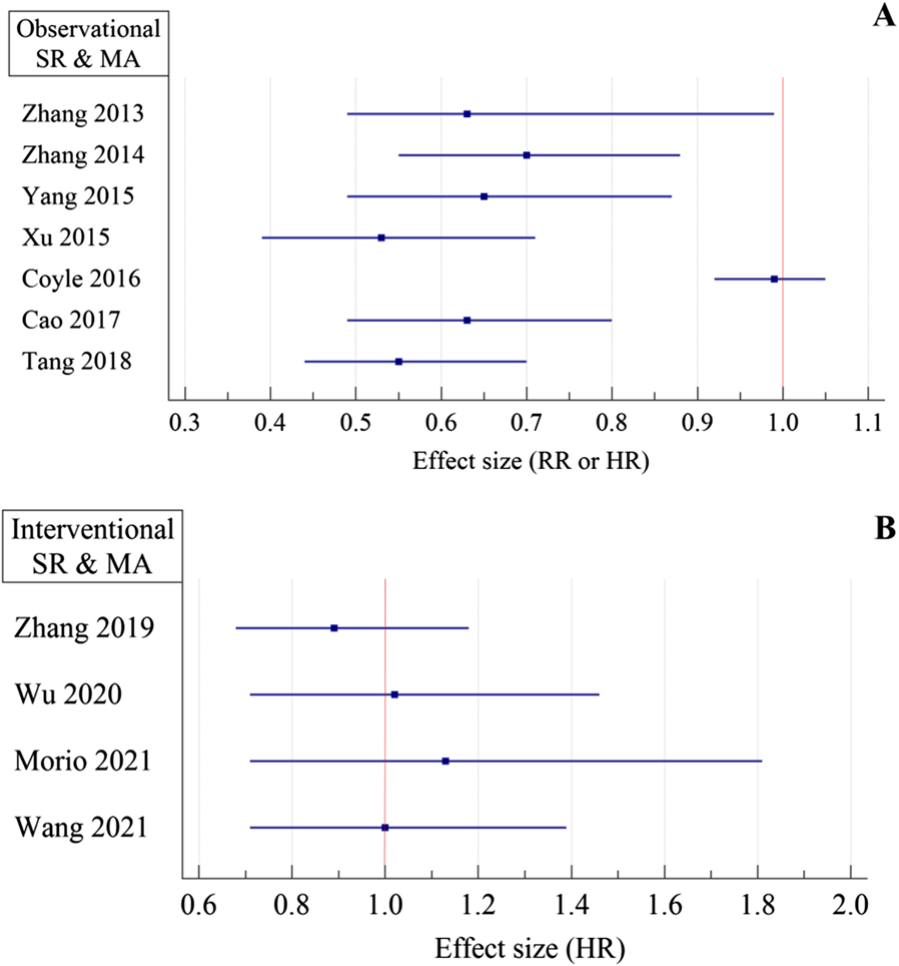
Observational meta-analyses on the association of metformin with all-cause mortality in BC patients. **A** metformin vs. non-metformin groups, **B** metformin vs. placebo, *RR* risk ratio, *HR* hazard ratio

**Fig. 5:**
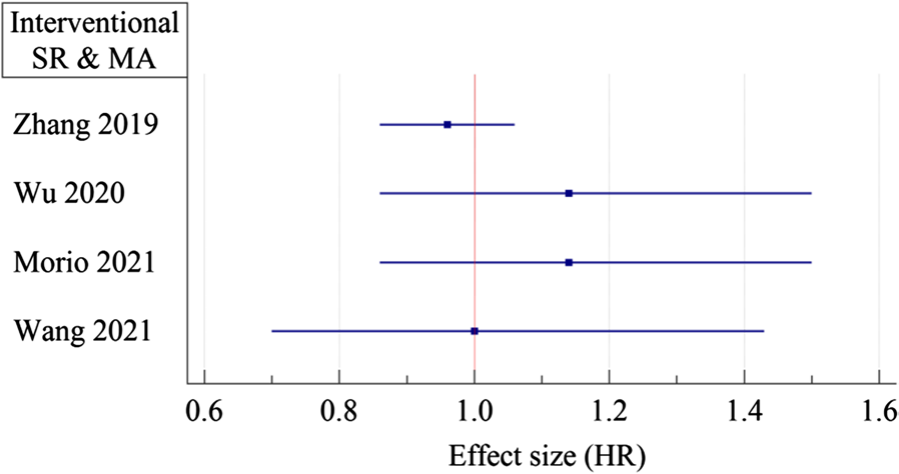
International meta-analyses on the association of metformin with progression-free survival of BC patients metformin vs. placebo. *HR* hazard ratio

In a systematic review and meta-analysis conducted by Tang et al., the protective effect of metformin against BC mortality in patients with type 2 diabetes has been assessed. An approximate total of 3400 metformin users and 3000 non-metformin users were included. In the metformin user group, they found a 45% lower risk for all-cause mortality (HR = 0.55; 95% CI 0.44 to 0.70). However overall certainty of the evidence was very low and the included studies were only observational studies [73]. In a similar study conducted by Xu et al., the BC-specific survival time was better in the metformin user group (HR: 0.89; 95% CI 0.79 to 1.00). In diabetic patients who used metformin after BC diagnosis, a 36% risk reduction for cancer-specific mortality was reported (HR = 0.64; 95% CI 0.45 to 0.90). Also in BC patients who consumed metformin, a significant risk reduction of all-cause mortality compared with their non-diabetic parallel was observed (HR = 0.63; 95% CI 0.51 to 0.78) [42]. Overall, most studies demonstrate that metformin reduces BC mortality, especially in early-stage cancers. Nonetheless, caution is in order, as all of these studies were observational, and the results were heterogeneous. Despite observational systematic reviews and meta-analyses indicating better outcomes for metformin than placebo in BC patients (Fig. 4A) [42, 73–78], the adjuvant role of metformin was never confirmed in interventional systematic reviews and meta-analyses (Figs. 4B and 5) [78–82].

In an interventional systematic review and meta-analysis conducted by Farkhondeh and colleagues for BC molecular markers, they discovered that metformin had no considerable effect on Ki-67 concentration (SMD = 0.08, 95% CI − 0.14 to 0.30) in the metformin group versus the placebo group [83]. In contrast, a similar study by Rahmani et al. showed a significant reduction in Ki-67 concentration (weighted MD = − 4.06, 95% CI − 7.59 to − 0.54) for the metformin group [84]. This inconsistency may relate to the different inclusion criteria of these studies.

Survival benefits of metformin in BC cohort studies have been reported in patients with positive ER or PR, HER2 overexpression, and high IGF-I receptor expression on the tumor cell surface [85]. According to a study conducted by Park et al*.* on 44,541 women, metformin was given to the majority of the diabetic population in the study (61%) as part of their treatment, they did not find any correlation between the use of metformin and the overall risk of BC after a median follow-up of 6–8 years. However, based on their findings, metformin therapy was associated with a reduced risk of estrogen receptor-positive (ER-positive) BC, and this inverse association was even more pronounced in a longer period (e.g., 10 years) treatment. The results of this study suggest that there is a T2D-dependent link between ER status and BC, and long-term metformin use might be able to reduce the association between T2D and ER-positive BC [86]. Also, an observational study from 23 Spanish hospitals found that metformin had a protective effect against ER-positive/HER2-negative BC when used as a daily treatment [87].

Finally, a large multicenter phase 3 RCT (the MA.32 RCT) for determining the adjuvant effect of metformin in patients with BC but without diabetes has been conducted recently. To the best of our knowledge, this study has the largest sample size for this topic even more than systematic reviews and meta-analyses (with 3649 enrolled patients and eight years of follow-up). The analysis demonstrated overall survival will not change with the addition of metformin vs placebo to the standard BC regimen (HR = 1.10; 95% CI 0.86 to 1.41; P = 0.47) [88].

## LncRNAs in BC

The term lncRNAs refers to RNA molecules that contain over 200 nucleotides without encoding proteins. Many lncRNAs have a 5′ cap that makes their RNA structure more stable. The polyadenylation of the 3′ end of the lncRNA can also affect the stabilization of the structure of the lncRNA, but this can only be observed at certain points in the molecule [89]. The H19 and Xist genes were the first identified lncRNAs, classified as mRNAs at the time of their discovery [90]. It has been shown that lncRNA with long exons contains a number of exon regions, which allows the creation of different forms of this RNA family by splicing them together. In addition to their different functions, these forms may perform a variety of clinical roles as well [89]. The structure of lncRNAs allows them to escape the evolutionary limitations associated with poor interspecies conservation. Furthermore, the low conservation of the lncRNA sequence is likely to allow the structure of the lncRNA to be variable, to activate lncRNA functions and specializations as regulators within the cell [91]. The fact that lncRNAs have a high degree of specificity can be attributed to their key role in regulating organism function as well as in repairing disease processes in different conditions [92].

Originally, lncRNAs were thought to be genomic noise without any biological function. Recently, researchers have begun focusing their attention on the role lncRNAs. The interaction between RNA–protein, RNA–RNA, and DNA–RNA can be associated with lncRNA and form different functional complexes. As a result of their ability to regulate mRNA stability, translation, and cell signaling pathways, lncRNA can perform a variety of functions within the cell [93]. LncRNAs can be classified into five groups based on their closest protein-coding transcripts: sense, antisense, bidirectional, intronic, and intergenic [9]. In general, lncRNAs act in four distinct ways as signal, decoy, guide, and scaffold molecules [94]. lncRNAs are often thought to regulate downstream gene transcription as signal molecules, for example, lincRNA-p21 regulates JAK2/STAT3, Notch signaling. Occasionally, lncRNAs can also act as a decoy molecule to block certain molecular pathways. lncRNAs can inhibit the expression of mRNA by interacting with certain proteins. Decoying transcriptional factors or miRNAs, RNA interference, targeting of transcriptional factors, or chromatin modifier proteins to specific genomic loci, and transcriptional regulation in cis or trans [16]. In order to regulate gene transcription, lncRNAs can also act as a guide molecule that interacts with transcription factors on specific sequences of DNA and recruit chromatin-modifying enzymes for target genes, chromatin remodeling, and their epigenetic regulation [16, 95]. In addition, lncRNAs as scaffold molecules facilitate the assembly of various kinds of macromolecular complexes, promoting information, integration, and convergence [96]. Regulation of stabilized ribonucleoprotein complexes such as signaling molecules and nuclear structures is considered among the scaffolding activities of lncRNAs. The expression of genes can be regulated by lncRNAs at several levels, including epigenetics, transcription, and post-transcriptional regulation [97]. Contrary to mRNA, lncRNAs are found throughout the cell, not only in the nucleus but also in the cytoplasm and mitochondria [98]. There has been evidence that diseases linked to single-nucleotide polymorphisms within lncRNA genes and their promoters are associated with change in lncRNA expression which highlights their significance in the pathogenesis of the disease. The lncRNAs act as guides, scaffolds, or stabilizers that affect chromatin architecture and gene expression through interactions with epigenetic remodelers, transcription factors, and spliceosomes in the nucleus [99]. As lncRNA decoys, cytoplasmic lncRNAs regulate the stability of mRNA by directly regulating de-adenylation [93]. Xist is an important example of nuclear lncRNA which play an important role in females’ X chromosome by directing methyltransferases to the X chromosome. Xist facilitates macrophage polarization in breast and ovarian cancer. This function could be due to the regulation of C/EBPα and KLF6 by miR-101 [100]. There have been numerous studies on the association of lncRNAs and different types of cancers which make lncRNAs interesting targets for unique therapeutic and diagnostic pathways. LncRNAs play important role in cancer by regulating transcription and chromatin remodeling through interactions with chromatin remodelers like polycomb complex. For example, in BC, lncRNA PANDAR plays a key role in G1 to S phase regulation [101]. Regarding metastatic breast carcinomas, HOTAIR has changed the pattern of PRC2 occupancy, causing it to shift from breast epithelial cells to embryonic fibroblasts due to its ability to alter chromatin [102].

Normal development of breast stem cells is driven by some of the same signaling pathways, including estrogen receptors, HER2, and Wnt/b-catenin signaling pathways that control stem cell proliferation, cell death, cell differentiation, and cell motility. Regulation of BC heterogeneity and plasticity is one of the most urgent issues for treatment. Studies confirm that epigenetic regulation and non-coding RNAs may play an important role in BC development and may contribute to the heterogeneous and metastatic aspects of BC, especially triple-negative BC [11]. Many studies have investigated the association between lncRNAs and BC. In addition to basic research, the clinical application of lncRNAs is also an emerging research field [16]. lncRNAs expressed in cancers play essential roles in cancer-related biological processes and signaling pathways, regulating gene expression, post-transcriptional processes, chromatin changes, and regulation of protein function. Studies show that the distribution and activity of lncRNAs and their role in human cancers can be confirmed by transcription profiling studies [103]. Most of the activity of lncRNA is related to transcription. These activities include the formation of chromatin-modifying complexes, transcription activators, and chromatin ring regulators. Regulating the transcription of tumor suppressor and oncogene genes is one of the functions related to lncRNA oncology [103]. Some lncRNAs target DICER or pre-miRNA and hinder miRNA biogenesis that influences BC metastasis. For example overexpression of oncogenic lncRNA LINC01787 promotes BC cell proliferation, migration, and BC xenograft growth in vivo by repressing the maturation of miR-125b. The upregulation of LINC00899 represses the proliferation, migration, and invasion of BC cells by inhibiting miR-425 [9]. One of the main causes of death associated with BC is the metastasis of the primary tumor. lncRNAs are additional transcripts related to metastasis and cancer progression. Due to the necessary advances in transcriptome analysis technology, many articles confirm the expression of lncRNA in tumors and their correlation with metastatic conditions [104]. Since lncRNAs have a variable expression in cancer tissues compared to normal tissues, it raises the potential of these molecules as biomarkers for disease diagnosis. lncRNAs can alter multiple signaling pathways and regulate metastasis-related factors, alter the proteins, and transcription factors involved in metastasis. They can be used as an early diagnostic and therapeutic target for BC metastasis and therapy. For example, anti-metastatic lncRNAs can target oncogenes and inhibit metastasis whereas some prometastatic lncRNA reduce the expression of tumor suppressor genes and induce invasion and metastasis. Antisense oligonucleotides (ASOs) can reduce the expression of oncogenic lncRNAs and inhibit BC metastasis by degrading lncRNAs, cleaving endogenous RNaseH1, or regulating RNA–protein interactions [9]. Different novel technologies target lncRNAs in cancer therapy by small molecule inhibitors which are new directions in anti-tumor drug development, including targeting cytoplasmic and nuclear lncRNA by ASOs through RNaseH-dependent degradation to knock out lncRNAs, nanomedicine role of lncRNAs is associated with nano-carrier-absorbed on nano-drugs that target sub-nucleus lncRNAs to gain desired therapeutic effect and in cancer cell chemical resistance to different types of drugs. Other approaches include knock-out of lncRNA via specific gDNA by CRISPR/cas9 as a technology with extensive application in cancer treatment, and finally targeting lncRNAs by virus therapies including encapsulated shRNA to target tumor suppressor lncRNAs or natural antisense RNAs (NATs)-mediated technique [105, 106].

Generally, BC cells associated lncRNAs can be classified into two groups of enhance or attenuate the aggressiveness of BC cells, for example, DANCR, H19, HOTAIR, LINC00152, LINC00461, NEAT1, and LINC01857 cause invasion and migration of BC cells, H19, HOTAIR, HIF1A-AS2, RP1, and MALAT1 promote distant metastasis of BC cells, GAS5, MT1JP, NEF, NKILA, LET, TFAP2A-AS1, LncKLHDC7B prevent the invasion and migration of BC cells, MALAT1, MEG3, NLIPMT, and XIST inhibit distant metastasis of BC cells [16]. Also, in some studies, immune-related lncRNA signatures were associated with survival of BC [107–109], or the significant dysregulation of lncRNAs in BC was not associated with any of the clinical features [110]. Our study investigated the role of lncRNAs on BC in more than 40 general effects including metastasis, migration, invasion, progression, tumor growth, apoptosis, stemness, EMT, survival, histological grade, tumor size, tumor stage, proliferation, chemoresistance, radioresistance, drug resistance, endocrine resistance, disease recurrence, viability, differentiation, tumor initiation, aggressiveness, cell cycle, prognosis, diagnosis, tumor values, steroid receptor status, hormone negativity, tumourigenesis, colonization, invasion-metastasis cascade, colony formation, angiogenesis, motility, mobility, biological activities of BC cells, pathogenesis, clinicopathological features, clinical outcome, tumorigenic properties. Finally, 116 lncRNAs associated with these BC features were identified. In general most of lncRNAs were associated with invasion, migration, metastasis, and proliferation. The detailed results are presented in Table 1 and Additional file 1: Table S1.

## Metformin action on lncRNAs in BC

Some of the lncRNAs listed in Table 1 including GAS5, HOTAIR, TUG1, MALAT1, and H19 are also associated with the effect of metformin on BC. As described in the antitumor mechanisms of metformin, it effects on mTOR signaling pathway. The inhibition of the mTOR is responsible for regulating tumor growth [48]. mTOR is related to liver kinase B1 (LKB1), a major downstream kinase of AMPK. There has been evidence that deletion of LKB1 function in tumor cells accelerated their proliferation and resulted in them becoming more sensitive to AMPK pathway activators, such as biguanide (metformin) in both in vitro and in vivo studies [227, 228]. Metformin inhibits the over-activation of this pathway through upregulating lncRNA GAS5 expression, and finally induces apoptosis and inhibits the growth of BC cells [229]. As GAS5 is associated with invasion, growth, tumor promotion, proliferation, and apoptosis of BC (Table 1 and Additional file 1: Table S1), metformin can regulate these features by GAS5 antitumor activity. Metformin reverses EMT by inducing DNA methylation of the CpG-rich sequence at the gene downstream region in HOTAIR and downregulating the HOTAIR oncogenic expression in MDA-MB-231 BC cells [230]. The HOTAIR is associated with progression, metastasis, prognosis, cell growth, migration, invasion, apoptosis, and EMT in BC (Table 1 and Additional file 1: Table S1), so metformin can regulate these features by changing HOTAIR expression. Crosstalk between autophagy and Wnt/β-catenin signaling is frequent and is directly related to cell homeostasis [231]. There is also strong evidence from the current studies that inhibiting EMT results in the inactivation of Wnt/β-catenin, a primary mediator of EMT [232]. In breast cancer cells, metformin might also inhibit cell migration by inactivating Wnt/β-catenin [233]. Metformin treatment resulted in elevated endoplasmic reticulum stress, which was further intensified by knocking down MALAT1 expression [234]. MALAT1 is involved in metformin inhibitory role in the BC cells proliferation. Metformin increases the expression of HOTAIR, MALAT1, TUG1, LINC01121, and DICER1-AS1 in BC cells. MALAT1 knock-down in metformin-treated BC cells will increase the Bax/Bcl2 ratio and p21, Beclin1, VDAC1, LC3-II, CHOP and Bip expressions will decrease cyclin B1 [62]. As MALAT1 and TUG1 are associated with migration, invasion, metastasis, progression, EMT, relapse-free survival, proliferation, angiogenesis, motility, apoptosis, tumor growth, and tumor size of BC (Table 1 and Additional file 1: Table S1), metformin can regulate these features by MALAT1 and TUG1 activities. H19 is a transcription product of the H19 gene. H19 potentially influences gene expression in BC on several levels, including epigenetic regulation, transcriptional regulation, and posttranscriptional regulation. In BC tumorigenesis and progression, abnormal expression of H19 is governed by a number of molecular mechanisms, including microRNA-675 encoding, competing with endogenous RNA regulation, and interacting with Myc [235, 236]. Angiogenesis, cell death, inflammation, and apoptosis are some of the features that H19 stimulates [237]. There is growing evidence that ferroptosis, an emerging form of cell death that suppresses drug resistance and enhances the immune system’s ability to combat tumors, could be considered a new form of programmed cell death. In terms of cell necrosis, ferroptosis is defined as a process that depends on iron [238]. In case of ferroptosis, the membrane structure can be damaged owing to the formation of lipid peroxidation, which results in oxidative damage to the phospholipids, and in addition, there can be a high concentration of unsaturated fatty acids in the membrane [239]. One of the most common symptoms of ferroptosis is the excess of reactive oxygen species (ROS), which can be caused by metformin [240]. Metformin may induce ferroptosis by inhibiting autophagy via lncRNA H19 in BC subjects [238]. The effect of metformin on H19 lead to change in proliferation, invasion, migration, metastasis, cell cycle arrest, apoptosis, steroid receptor status, tumor size, disease-free survival, prognosis, stemness, EMT, and MET features of BC patients.

lncRNAs UCA1, H19, MALAT1, AFAP1-AS1, AC026904.1, and SNHG7 presented in Table 1 are associated with the effect of metformin on other types of cancer. Metformin promotes apoptosis and inhibits the proliferation of colon cancer cells by inhibiting UCA1 expression [241]. Metformin inhibits tumor cell invasion and migration partly by H19 downregulation [242] and decreases the expression of H19 in Endometrial Cancer [243]. Metformin decreases migration and invasion of cervical cancer cells by suppressing MALAT1 and disrupting of MALAT1/miR-142-3p sponge [244]. Metformin suppresses lung adenocarcinoma by downregulating AFAP1-AS1 and regulating the AFAP1-AS1/miR-3163/SPP1/PI3K/Akt/mTOR axis. [245]. Metformin suppresses hypopharyngeal cancer growth by decreasing SNHG7 expression through activating SAHH [246].

Other lncRNAs including AC006160.1, Loc100506691, lncRNA-AF085935, and HULC are also associated with cancers and metformin. Bladder cancer patients with high expression of AC006160.1 are sensitive to metformin [247]. Metformin anti-proliferative effects in gastric cancer may be associated with suppression of Loc100506691 (an oncogenic lncRNA) and Loc100506691-miR-26a-5p/miR-330-5p-CHAC1 axis [248]. In HepG2cells, metformin and EGCG combination shows anticarcinogenic effects by changes in proliferation, lncRNA-AF085935 expression, and apoptosis [249]. Metformin reduces HULC overexpression to inhibit HBV-induced hepatocellular carcinoma tumorigenesis [250].

## Supplementary Information

**Additional file 1: Table S1.** lncRNAs associated with BC (continuation of Table 1).

## Data Availability

Not applicable.

## Abbreviations

BC: Breast cancer
lncRNA: Long non-coding RNAs
T2D: Type 2 diabetes
TNBC: Triple-negative breast cancer
HER2: Human epidermal growth factor receptor 2
MET: Mesenchymal-to-epithelial transition
EMT: Epithelial-to-mesenchymal transition
MMP-9: Matrix metalloproteinase 9
P53: Tumor protein 53
CTCs: Circulating tumor cells
piRNAs: P-element-induced wimpy testis interacting RNAs
ER: Estrogen receptor
PR: Progesterone receptor
mTOR: Mechanistic target of rapamycin
EGFR: Epidermal growth factor receptor
AMPK: AMP-activated protein kinase
MAPK: Mitogen-activated protein kinases
PKB: Phosphorylation of protein kinase B
GRB2: Growth factor receptor-bound protein 2
ERK: Signal-regulated kinase
NFκB: Nuclear factor kappa
STAT3: Signal transducer activator of transcription 3
HIF1α: Hypoxia-inducible factor 1-alpha
c-Myc: Cellular-Myc
DICER: Double-stranded RNA specific endoribonuclease
ACC: Acetyl-CoA carboxylase
FASN: Fatty acid synthase
IGFBP-3: Insulin-like growth factor binding protein 3
NATs: Natural antisense RNAs
ATG: Autophagy-related
ER-positive: Estrogen receptor-positive
ASOs: Antisense oligonucleotides
LKB1: Liver kinase B1
ROS: Reactive oxygen species
