# Supplementary material for: Metformin and long non-coding RNAs in breast cancer
Source: J Transl Med. 2023 Feb 27;21:155. doi: 10.1186/s12967-023-03909-x (PMC9969691; doi:10.1186/s12967-023-03909-x)
Supplement: Supplementary file 1 — Additional file 1: Table S1. lncRNAs associated with BC (continuation of Table 1). [file 12967_2023_3909_MOESM1_ESM.docx]

Table S1. lncRNAs associated with BC (continuation of Table 1)

| **LncRNA/ID (Ensemble/NCBI)** | **Dysregulation of lncRNA (Promote/Inhibit)** | **Oncogene/ Tumor Suppressor** | **Effect on BC** | **Ref.** |
| --- | --- | --- | --- | --- |
| AFAP1-AS1/ ENSG00000272620 | Upregulation/Promote | Oncogene | Overall survival, Disease-free survival, Progression-free survival, Lymph node metastasis, Distant metastasis, Histological grade, Larger tumor size, Tumor stage | [[1](#_ENREF_1)] |
|  |  |  | Proliferation, Invasion | [[2](#_ENREF_2)] |
|  |  |  | Progression, Invasion | [[3](#_ENREF_3)] |
| ANCR/ ENSG00000226950 | Upregulation/Inhibit | Tumor Suppressor | EMT, Metastasis, Migration, Invasion | [[4](#_ENREF_4)] |
| DANCR/ ENSG00000226950 | Upregulation/Promote | Oncogene | Proliferation, Invasion, Tumor growth | [[5](#_ENREF_5)] |
|  |  |  | Advanced tumor grades, Lymph node metastasis | [[6](#_ENREF_6)] |
| ATB (LNCRNA-ATB)/ Gene ID: 114004396 | Upregulation/Promote | Oncogene | EMT, Drug resistance, Invasion, Metastasis | [[7](#_ENREF_7)] |
| BC200 (BCYRN1)/ ENSG00000236824 | Upregulation/Promote | Oncogene | Proliferation, Survival, Tumor growth | [[8](#_ENREF_8)] |
|  |  |  | Survival, Proliferation | [[9](#_ENREF_9)] |
| BCAR4/ ENSG00000262117 | Upregulation/Promote | Oncogene | Aggressiveness, Drug resistance, Overall survival, Metastasis-free survival | [[10](#_ENREF_10)] |
|  |  |  | Endocrine resistance, Metastasis free survival, Overall survival | [[11](#_ENREF_11)] |
| BORG | Upregulation/Promote | Oncogene | Cell survival, Chemoresistance | [[12](#_ENREF_12)] |
| CASC2/ ENSG00000177640 | Upregulation/Inhibit | Tumor suppressor | Cell viability, Migration, Invasion, Apoptosis | [[13](#_ENREF_13)] |
|  |  |  | Drug resistance, Proliferation, Tumor growth | [[14](#_ENREF_14)] |
| CCAT1/ Gene ID: 100507056 | Upregulation/Promote | Oncogene | Differentiation grade, TNM stage, Lymph node metastases, Overall survival, Progression-free survival, Progression-free survival | [[15](#_ENREF_15)] |
| CCAT2/ ENSG00000280997 | Upregulation/Promote | Oncogene | Invasion, Metastasis | [[16](#_ENREF_16)] |
|  |  |  | Tumor initiation, Progression, Stemness, Aggressiveness | [[17](#_ENREF_17)] |
|  |  |  | Chemo-sensitivity | [[18](#_ENREF_18)] |
|  |  |  | Proliferation, invasion | [[19](#_ENREF_19)] |
|  |  |  | Tumor growth, Tumor formation | [[20](#_ENREF_20)] |
| EPIC1/ ENSG00000224271 | Upregulation/Promote | Oncogene | Drug resistance | [[21](#_ENREF_21)] |
| GACAT3/ ENSG00000236289 | Upregulation/Promote | Oncogene | Proliferation, Prognosis, | [[22](#_ENREF_22)] |
| GAS5/ ENSG00000234741 | Upregulation/Inhibit | Tumor suppressor | Invasion, Tumor growth | [[23](#_ENREF_23)] |
|  |  |  | Apoptosis | [[24](#_ENREF_24)] |
|  |  |  | Apoptosis, Drug resistance | [[25](#_ENREF_25)] |
|  |  |  | Apoptosis | [[26](#_ENREF_26)] |
|  |  |  | Drug resistance, Prognosis, Proliferation, Tumor growth, | [[27](#_ENREF_27)] |
|  |  |  | Drug resistance, Prognosis | [[28](#_ENREF_28)] |
|  |  |  | Prognosis | [[29](#_ENREF_29)] |
|  |  |  | Apoptosis, Tumor growth | [[30](#_ENREF_30)] |
|  |  |  | Apoptosis | [[31](#_ENREF_31)] |
|  |  |  | Drug resistance | [[32](#_ENREF_32)] |
|  |  |  | Tumor promotion, Proliferation, Apoptosis | [[33](#_ENREF_33)] |
| H19/ ENSG00000130600 | Upregulation/Promote | Oncogene | Tumor values, Steroid receptor status | [[34](#_ENREF_34)] |
|  |  |  | Tumor size, Nodal status, Disease-free survival, Prognosis, Stemness, Hormone | [[35](#_ENREF_35)] |
|  |  |  | Migration, Invasion, Metastasis | [[36](#_ENREF_36)] |
|  |  |  | Invasion, Proliferation | [[37](#_ENREF_37)] |
|  |  |  | Metastasis, MET, EMT | [[38](#_ENREF_38)] |
|  |  |  | Stemness | [[39](#_ENREF_39)] |
|  |  |  | Drug resistance, Apoptosis | [[40](#_ENREF_40)] |
|  |  |  | Tumorigenic properties, Tumor growth | [[41](#_ENREF_41)] |
|  |  |  | Tumourigenesis | [[42](#_ENREF_42)] |
|  |  |  | Proliferation, Survival | [[43](#_ENREF_43)] |
|  |  |  | Proliferation, Differentiation | [[44](#_ENREF_44)] |
|  |  |  | Progression | [[45](#_ENREF_45)] |
|  |  |  | Drug resistance, Apoptosis | [[46](#_ENREF_46)] |
|  |  |  | Drug resistance, Cell cycle arrest | [[47](#_ENREF_47)] |
|  |  |  | Drug resistance | [[48](#_ENREF_48)] |
|  |  |  | Migration, Invasion, Metastasis, EMT | [[49](#_ENREF_49)] |
|  |  |  | Drug resistance | [[50](#_ENREF_50)] |
| HIF1A-AS2/  Gene ID: 100750247 | Upregulation/Promote | Oncogene | Recurrence-free survival | [[51](#_ENREF_51)] |
|  |  |  | Proliferation, Invasion, Drug resistance | [[52](#_ENREF_52)] |
| HOST2 (CERNA2)/ ENSG00000285972 | Upregulation/Promote | Oncogene | Proliferation, Migration | [[53](#_ENREF_53)] |
| HOTAIR/ ENSG00000228630 | Upregulation/Promote | Oncogene | Invasion, Metastasis | [[54](#_ENREF_54)] |
|  |  |  | Invasion, Progression, Metastasis, Prognosis | [[55](#_ENREF_55)] |
|  |  |  | Invasion, Migration, Survival, Prognosis | [[56](#_ENREF_56)] |
|  |  |  | Proliferation, Invasion, Migration | [[57](#_ENREF_57)] |
|  |  |  | Tumor growth, Metastasis | [[58](#_ENREF_58)] |
|  |  |  | Invasion, Metastasis | [[59](#_ENREF_59)] |
|  |  |  | Metastasis | [[60](#_ENREF_60)] |
|  |  |  | Proliferation, Metastasis | [[61](#_ENREF_61)] |
|  |  |  | Tumorigenesis, Proliferation, Drug resistance | [[62](#_ENREF_62)] |
|  |  |  | Cell growth, Migration, Invasion, Apoptosis | [[63](#_ENREF_63)] |
|  |  |  | EMT | [[64](#_ENREF_64)] |
|  |  |  | Proliferation, Colony formation | [[65](#_ENREF_65)] |
|  |  |  | Proliferation, Migration, Invasion, Tumor growth | [[66](#_ENREF_66)] |
|  |  |  | Proliferation, Migration, Invasion, EMT , Tumor growth | [[67](#_ENREF_67)] |
|  |  |  | Migration | [[68](#_ENREF_68)] |
|  |  |  | Radioresistance | [[69](#_ENREF_69)] |
| LINC00152 (CYTOR)/ ENSG00000222041 | Upregulation/Promote | Oncogene | Drug resistance | [[70](#_ENREF_70)] |
| LINC00511/ Gene ID: 400619 | Upregulation/Promote | Oncogene | Cytotoxicity | [[71](#_ENREF_71)] |
| Linc-ROR/ ENSG00000258609 | Upregulation/Promote | Oncogene | Tumorigenesis | [[72](#_ENREF_72)] |
|  |  |  | Progression | [[73](#_ENREF_73)] |
|  |  |  | Invasion | [[74](#_ENREF_74)] |
|  |  |  | Prognostic outcomes, Proliferation, Invasion, Progression, Tumor growth | [[75](#_ENREF_75)] |
|  |  |  | Drug resistance | [[76](#_ENREF_76)] |
|  |  |  | Tumor growth, Drug resistance, Outcomes | [[77](#_ENREF_77)] |
|  |  |  | Drug resistance, Invasion, EMT | [[78](#_ENREF_78)] |
| LINP1/ ENSG00000223784 | Upregulation/Promote | Oncogene | Migration, Invasion, Metastasis, Chemoresistance | [[79](#_ENREF_79)] |
|  |  |  | Lymph node Metastasis, Differentiation, Survival, Disease-free survival | [[80](#_ENREF_80)] |
| MAGI2-AS3/ ENSG00000234456 | Upregulation/Inhibit | Tumor Suppressor | Metastatic progression, Migration, Invasion | [[81](#_ENREF_81)] |
| MALAT1/ ENSG00000251562 | Upregulation/Inhibit | Tumor Suppressor | EMT, Relapse-free survival | [[82](#_ENREF_82)] |
|  |  |  | Metastasis, Progression | [[83](#_ENREF_83)] |
|  | Upregulation/Promote | Oncogene | Migration, Invasion | [[84](#_ENREF_84)] |
|  |  |  | Proliferation, Migration, Angiogenesis | [[85](#_ENREF_85)] |
|  |  |  | Proliferation, Motility, Apoptosis, Tumor growth, Metastasis | [[86](#_ENREF_86)] |
|  |  |  | Proliferation, Migration, Invasion, Progression | [[87](#_ENREF_87)] |
|  |  |  | Proliferation, Apoptosis, Motility | [[88](#_ENREF_88)] |
|  |  |  | Proliferation, Migration, Invasion, Apoptosis, Cell cycle arrest | [[89](#_ENREF_89)] |
|  |  |  | Invasion, EMT, Diagnosis | [[90](#_ENREF_90)] |
|  |  |  | Proliferation | [[91](#_ENREF_91)] |
|  |  |  | Recurrence, metastasis, Invasion, Migration | [[92](#_ENREF_92)] |
| MAYA (MNX1-AS1)/ ENSG00000243479 | Upregulation/Promote | Oncogene | Survival, Aggressiveness | [[93](#_ENREF_93)] |
| MEG3/ ENSG00000214548 | Upregulation/Inhibit | Tumor Suppressor | Proliferation, EMT, Invasion | [[94](#_ENREF_94)] |
|  |  |  | Tumor growth, Angiogenesis, Proliferation, Migration | [[95](#_ENREF_95)] |
|  |  |  | Metastasis, Prognosis, Differentiation | [[96](#_ENREF_96)] |
|  |  |  | Metastasis, Stage, Prognosis | [[97](#_ENREF_97)] |
| MT1JP/ ENSG00000255986 | Upregulation/Inhibit | Tumor Suppressor | Biological activities of breast cancer cells | [[98](#_ENREF_98)] |
|  |  |  | Proliferation, Migration, Invasion | [[99](#_ENREF_99)] |
| NEAT1/ ENSG00000245532 | Upregulation/ Promote | Oncogene | Metastasis, EMT, Invasion, Histological grades, Prognosis | [[100](#_ENREF_100)] |
|  |  |  | Metastasis | [[101](#_ENREF_101)] |
|  |  |  | Invasion, EMT, Proliferation, Apoptosis, Tumor growth | [[102](#_ENREF_102)] |
|  |  |  | Prognosis, Proliferation, Invasion, Tumorigenesis, Progression | [[103](#_ENREF_103)] |
|  |  |  | Apoptosis, Proliferation, Colony formation, Tumor growth, Drug resistance, Chemoresistance | [[104](#_ENREF_104)] |
|  |  |  | Cell growth, Migration, Invasion | [[105](#_ENREF_105)] |
| NKILA/ ENSG00000278709 | Upregulation/Inhibit | Tumor Suppressor | Metastasis, Prognosis | [[106](#_ENREF_106)] |
| PTENP1/ ENSG00000237984 | Upregulation/Inhibit | Tumor Suppressor | Cell survival, Colony forming, Migration, Invasion, Apoptosis | [[107](#_ENREF_107)] |
|  |  |  | Proliferation, Migration | [[108](#_ENREF_108)] |
|  |  |  | Metastasis, Cell growth, Progression | [[109](#_ENREF_109)] |
| SPRY4-IT1/GeneID:100642175 | Upregulation/Promote | Oncogene | Pathogenesis | [[110](#_ENREF_110)] |
| TINCR/ ENSG00000223573 | Upregulation/Promote | Oncogene | Overall survival, Clinicopathological features, Clinical outcome | [[111](#_ENREF_111)] |
| UCA1/ ENSG00000214049 | Upregulation/Promote | Oncogene | Invasion-metastasis cascade, EMT | [[112](#_ENREF_112)] |
|  |  |  | EMT, Invasion | [[113](#_ENREF_113)] |
|  |  |  | Proliferation | [[114](#_ENREF_114)] |
|  |  |  | Drug resistance | [[115](#_ENREF_115)] |
|  |  |  | Drug resistance | [[116](#_ENREF_116)] |
|  |  |  | Drug resistance | [[117](#_ENREF_117)] |
|  |  |  | Drug resistance | [[118](#_ENREF_118)] |
|  |  |  | Metastasis | [[119](#_ENREF_119)] |
| XIST/ ENSG00000229807 | Downregulation/Promote | Tumor Suppressor | Prognosis, Tumor size, Overall survival, Disease-free survival, Metastasis | [[120](#_ENREF_120)] |
|  |  |  | Tumor growth, Stemness, Metastasis | [[121](#_ENREF_121)] |
|  |  |  | Cell viability | [[122](#_ENREF_122)] |
| TP73-AS1/ ENSG00000227372 | Upregulation/Promote | Oncogene | Apoptosis, Proliferation | [[123](#_ENREF_123)] |
| DSCAM-AS1/ ENSG00000235123 | Downregulation/ Inhibit | Oncogene | Apoptosis. Drug resistance | [[124](#_ENREF_124)] |
| SNHG7/ ENSG00000233016 | Upregulation/Promote | Oncogene | Drug resistance | [[125](#_ENREF_125)] |

**References**

1. Liu R-H, Wang M-Y, Chen L-Y, Yin Z-J, Ji Q-K, Wang Y-Y, et al. Meta-analysis of the prognostic value of long non-coding RNA AFAP1-AS1 for cancer patients in China. Oncotarget. 2018;9(8):8100.

2. Zhang X, Zhou Y, Mao F, Lin Y, Shen S, Sun Q. lncRNA AFAP1-AS1 promotes triple negative breast cancer cell proliferation and invasion via targeting miR-145 to regulate MTH1 expression. Scientific reports. 2020;10(1):1-11.

3. Zhang X, Li F, Zhou Y, Mao F, Lin Y, Shen S, et al. Long noncoding RNA AFAP1-AS1 promotes tumor progression and invasion by regulating the miR-2110/Sp1 axis in triple-negative breast cancer. Cell Death & Disease. 2021;12(7):1-11.

4. Li Z, Dong M, Fan D, Hou P, Li H, Liu L, et al. LncRNA ANCR down-regulation promotes TGF-β-induced EMT and metastasis in breast cancer. Oncotarget. 2017;8(40):67329.

5. Sha S, Yuan D, Liu Y, Han B, Zhong N. Targeting long non-coding RNA DANCR inhibits triple negative breast cancer progression. Biology Open. 2017;6(9):1310-6.

6. Zhang KJ, Tan XL, Guo L. The long non‐coding RNA DANCR regulates the inflammatory phenotype of breast cancer cells and promotes breast cancer progression via EZH2‐dependent suppression of SOCS3 transcription. Molecular oncology. 2020;14(2):309-28.

7. Shi S-J, Wang L-J, Yu B, Li Y-H, Jin Y, Bai X-Z. LncRNA-ATB promotes trastuzumab resistance and invasion-metastasis cascade in breast cancer. Oncotarget. 2015;6(13):11652.

8. Singh R, Gupta S, Peng W, Zhou N, Pochampally R, Atfi A, et al. Regulation of alternative splicing of Bcl-x by BC200 contributes to breast cancer pathogenesis. Cell death & disease. 2016;7(6):e2262-e.

9. Booy EP, McRae EK, Koul A, Lin F, McKenna SA. The long non-coding RNA BC200 (BCYRN1) is critical for cancer cell survival and proliferation. Molecular cancer. 2017;16(1):1-15.

10. Godinho MF, Sieuwerts A, Look M, Meijer D, Foekens J, Dorssers L, et al. Relevance of BCAR4 in tamoxifen resistance and tumour aggressiveness of human breast cancer. British journal of cancer. 2010;103(8):1284-91.

11. Godinho M, Meijer D, Setyono‐Han B, Dorssers LC, van Agthoven T. Characterization of BCAR4, a novel oncogene causing endocrine resistance in human breast cancer cells. Journal of cellular physiology. 2011;226(7):1741-9.

12. Gooding AJ, Zhang B, Gunawardane L, Beard A, Valadkhan S, Schiemann WP. The lncRNA BORG facilitates the survival and chemoresistance of triple-negative breast cancers. Oncogene. 2019;38(12):2020-41.

13. Gao Z, Wang H, Li H, Li M, Wang J, Zhang W, et al. Long non-coding RNA CASC2 inhibits breast cancer cell growth and metastasis through the regulation of the miR-96-5p/SYVN1 pathway. International Journal of Oncology. 2018;53(5):2081-90.

14. Zheng P, Dong L, Zhang B, Dai J, Zhang Y, Wang Y, et al. Long noncoding RNA CASC2 promotes paclitaxel resistance in breast cancer through regulation of miR-18a-5p/CDK19. Histochemistry and cell biology. 2019;152(4):281-91.

15. Zhang X-F, Liu T, Li Y, Li S. Overexpression of long non-coding RNA CCAT1 is a novel biomarker of poor prognosis in patients with breast cancer. International journal of clinical and experimental pathology. 2015;8(8):9440.

16. Redis RS, Sieuwerts AM, Look MP, Tudoran O, Ivan C, Spizzo R, et al. CCAT2, a novel long non-coding RNA in breast cancer: expression study and clinical correlations. Oncotarget. 2013;4(10):1748.

17. Xu Z, Liu C, Zhao Q, Lü J, Ding X, Luo A, et al. Long non-coding RNA CCAT2 promotes oncogenesis in triple-negative breast cancer by regulating stemness of cancer cells. Pharmacological research. 2020;152:104628.

18. Zhou D, Gu J, Wang Y, Luo B, Feng M, Wang X. Long noncoding RNA CCAT2 reduces chemosensitivity to 5‐fluorouracil in breast cancer cells by activating the mTOR axis. Journal of Cellular and Molecular Medicine. 2022;26(5):1392-401.

19. Deng X, Zhao Y, Wu X, Song G. Upregulation of CCAT2 promotes cell proliferation by repressing the P15 in breast cancer. Biomedicine & Pharmacotherapy. 2017;91:1160-6.

20. Cai Y, He J, Zhang D. Long noncoding RNA CCAT2 promotes breast tumor growth by regulating the Wnt signaling pathway. OncoTargets and therapy. 2015;8:2657.

21. Wang Y, Zhang M, Wang Z, Guo W, Yang D. MYC‐binding lncRNA EPIC1 promotes AKT‐mTORC1 signaling and rapamycin resistance in breast and ovarian cancer. Molecular carcinogenesis. 2020;59(10):1188-98.

22. Zhong H, Yang J, Zhang B, Wang X, Pei L, Zhang L, et al. LncRNA GACAT3 predicts poor prognosis and promotes cell proliferation in breast cancer through regulation of miR-497/CCND2. Cancer Biomarkers. 2018;22(4):787-97.

23. Zhang Z, Zhu Z, Watabe K, Zhang X, Bai C, Xu M, et al. Negative regulation of lncRNA GAS5 by miR-21. Cell Death & Differentiation. 2013;20(11):1558-68.

24. Filippova EA, Fridman MV, Burdennyy AM, Loginov VI, Pronina IV, Lukina SS, et al. Long noncoding RNA GAS5 in breast cancer: Epigenetic mechanisms and biological functions. International Journal of Molecular Sciences. 2021;22(13):6810.

25. Zheng S, Li M, Miao K, Xu H. lncRNA GAS5‐promoted apoptosis in triple‐negative breast cancer by targeting miR‐378a‐5p/SUFU signaling. Journal of cellular biochemistry. 2020;121(3):2225-35.

26. Pickard MR, Williams GT. Regulation of apoptosis by long non-coding RNA GAS5 in breast cancer cells: implications for chemotherapy. Breast cancer research and treatment. 2014;145(2):359-70.

27. Li W, Zhai L, Wang H, Liu C, Zhang J, Chen W, et al. Downregulation of LncRNA GAS5 causes trastuzumab resistance in breast cancer. Oncotarget. 2016;7(19):27778.

28. Gu J, Wang Y, Wang X, Zhou D, Shao C, Zhou M, et al. Downregulation of lncRNA GAS5 confers tamoxifen resistance by activating miR-222 in breast cancer. Cancer letters. 2018;434:1-10.

29. Ma C, Shi X, Zhu Q, Li Q, Liu Y, Yao Y, et al. The growth arrest-specific transcript 5 (GAS5): a pivotal tumor suppressor long noncoding RNA in human cancers. Tumor Biology. 2016;37(2):1437-44.

30. Mourtada-Maarabouni M, Pickard M, Hedge V, Farzaneh F, Williams G. GAS5, a non-protein-coding RNA, controls apoptosis and is downregulated in breast cancer. Oncogene. 2009;28(2):195-208.

31. Williams G, Pickard M. The hormone response element mimic sequence of GAS5 lncRNA is sufficient to induce apoptosis in breast cancer cells. Oncotarget. 2016;7(9).

32. Chen Z, Pan T, Jiang D, Jin L, Geng Y, Feng X, et al. The lncRNA-GAS5/miR-221-3p/DKK2 axis modulates ABCB1-mediated adriamycin resistance of breast cancer via the Wnt/β-catenin signaling pathway. Molecular Therapy-Nucleic Acids. 2020;19:1434-48.

33. Li S, Zhou J, Wang Z, Wang P, Gao X, Wang Y. Long noncoding RNA GAS5 suppresses triple negative breast cancer progression through inhibition of proliferation and invasion by competitively binding miR-196a-5p. Biomedicine & Pharmacotherapy. 2018;104:451-7.

34. Adriaenssens E, Dumont L, Lottin S, Bolle D, Leprêtre A, Delobelle A, et al. H19 overexpression in breast adenocarcinoma stromal cells is associated with tumor values and steroid receptor status but independent of p53 and Ki-67 expression. The American journal of pathology. 1998;153(5):1597-607.

35. Shima H, Kida K, Adachi S, Yamada A, Sugae S, Narui K, et al. Lnc RNA H19 is associated with poor prognosis in breast cancer patients and promotes cancer stemness. Breast cancer research and treatment. 2018;170(3):507-16.

36. Matouk IJ, Raveh E, Abu-lail R, Mezan S, Gilon M, Gershtain E, et al. Oncofetal H19 RNA promotes tumor metastasis. Biochimica et Biophysica Acta (BBA)-Molecular Cell Research. 2014;1843(7):1414-26.

37. Li Z, Li Y, Li Y, Ren K, Li X, Han X, et al. Long non‐coding RNA H19 promotes the proliferation and invasion of breast cancer through upregulating DNMT1 expression by sponging miR‐152. Journal of biochemical and molecular toxicology. 2017;31(9):e21933.

38. Zhou W, Ye X-l, Xu J, Cao M-G, Fang Z-Y, Li L-Y, et al. The lncRNA H19 mediates breast cancer cell plasticity during EMT and MET plasticity by differentially sponging miR-200b/c and let-7b. Science signaling. 2017;10(483):eaak9557.

39. Peng F, Wang J, Fan W, Meng Y, Li M, Li T, et al. Glycolysis gatekeeper PDK1 reprograms breast cancer stem cells under hypoxia. Oncogene. 2018;37(8):1062-74.

40. Si X, Zang R, Zhang E, Liu Y, Shi X, Zhang E, et al. LncRNA H19 confers chemoresistance in ERα-positive breast cancer through epigenetic silencing of the pro-apoptotic gene BIK. Oncotarget. 2016;7(49):81452.

41. Matouk IJ, DeGroot N, Mezan S, Ayesh S, Abu-lail R, Hochberg A, et al. The H19 non-coding RNA is essential for human tumor growth. PloS one. 2007;2(9):e845.

42. Zhang Z, Weaver DL, Olsen D, deKay J, Peng Z, Ashikaga T, et al. Long non-coding RNA chromogenic in situ hybridisation signal pattern correlation with breast tumour pathology. Journal of clinical pathology. 2016;69(1):76-81.

43. Basak P, Chatterjee S, Weger S, Bruce MC, Murphy LC, Raouf A. Estrogen regulates luminal progenitor cell differentiation through H19 gene expression. Endocrine-related cancer. 2015;22(4):505.

44. Sun H, Wang G, Peng Y, Zeng Y, Zhu Q-N, Li T-L, et al. H19 lncRNA mediates 17β-estradiol-induced cell proliferation in MCF-7 breast cancer cells. Oncology reports. 2015;33(6):3045-52.

45. Lottin S, Adriaenssens E, Dupressoir T, Berteaux N, Montpellier C, Coll J, et al. Overexpression of an ectopic H19 gene enhances the tumorigenic properties of breast cancer cells. Carcinogenesis. 2002;23(11):1885-95.

46. Han J, Han B, Wu X, Hao J, Dong X, Shen Q, et al. Knockdown of lncRNA H19 restores chemo-sensitivity in paclitaxel-resistant triple-negative breast cancer through triggering apoptosis and regulating Akt signaling pathway. Toxicology and applied pharmacology. 2018;359:55-61.

47. Wang J, Xie S, Yang J, Xiong H, Jia Y, Zhou Y, et al. The long noncoding RNA H19 promotes tamoxifen resistance in breast cancer via autophagy. Journal of hematology & oncology. 2019;12(1):1-14.

48. Zhu Q-N, Wang G, Guo Y, Peng Y, Zhang R, Deng J-L, et al. LncRNA H19 is a major mediator of doxorubicin chemoresistance in breast cancer cells through a cullin4A-MDR1 pathway. Oncotarget. 2017;8(54):91990.

49. Yan L, Yang S, Yue CX, Wei XY, Peng W, Dong ZY, et al. Long noncoding RNA H19 acts as a miR‐340‐3p sponge to promote epithelial‐mesenchymal transition by regulating YWHAZ expression in paclitaxel‐resistant breast cancer cells. Environmental toxicology. 2020;35(9):1015-28.

50. Basak P, Chatterjee S, Bhat V, Su A, Jin H, Lee-Wing V, et al. Long non-coding RNA H19 acts as an estrogen receptor modulator that is required for endocrine therapy resistance in ER+ breast cancer cells. Cellular Physiology and Biochemistry. 2018;51(4):1518-32.

51. Li J-w, Zhou J, Shi Z-t, Li N, Zhou S-c, Chang C. Sonographic Features of Triple-Negative Breast Carcinomas Are Correlated With mRNA–lncRNA Signatures and Risk of Tumor Recurrence. Frontiers in Oncology. 2021;10:587422.

52. Jiang Y-Z, Liu Y-R, Xu X-E, Jin X, Hu X, Yu K-D, et al. Transcriptome analysis of triple-negative breast cancer reveals an integrated mRNA-lncRNA signature with predictive and prognostic value. Cancer research. 2016;76(8):2105-14.

53. Hua K, Deng X, Hu J, Ji C, Yu Y, Li J, et al. Long noncoding RNA HOST2, working as a competitive endogenous RNA, promotes STAT3-mediated cell proliferation and migration via decoying of let-7b in triple-negative breast cancer. Journal of Experimental & Clinical Cancer Research. 2020;39(1):1-13.

54. Gupta RA, Shah N, Wang KC, Kim J, Horlings HM, Wong DJ, et al. Long non-coding RNA HOTAIR reprograms chromatin state to promote cancer metastasis. nature. 2010;464(7291):1071-6.

55. Wu Y, Zhang L, Wang Y, Li H, Ren X, Wei F, et al. Long noncoding RNA HOTAIR involvement in cancer. Tumor Biology. 2014;35(10):9531-8.

56. Liang H, Huang W, Wang Y, Ding L, Zeng L. Overexpression of MiR-146a-5p upregulates lncRNA HOTAIR in triple-negative breast cancer cells and predicts poor prognosis. Technology in Cancer Research & Treatment. 2019;18:1533033819882949.

57. Tao S, He H, Chen Q. Estradiol induces HOTAIR levels via GPER-mediated miR-148a inhibition in breast cancer. Journal of translational medicine. 2015;13(1):1-8.

58. Wang Y-L, Overstreet A-M, Chen M-S, Wang J, Zhao H-J, Ho P-C, et al. Combined inhibition of EGFR and c-ABL suppresses the growth of triple-negative breast cancer growth through inhibition of HOTAIR. Oncotarget. 2015;6(13):11150.

59. Zhang H, Cai K, Wang J, Wang X, Cheng K, Shi F, et al. MiR-7, inhibited indirectly by lincRNA HOTAIR, directly inhibits SETDB1 and reverses the EMT of breast cancer stem cells by downregulating the STAT3 pathway. Stem cells. 2014;32(11):2858-68.

60. Sørensen KP, Thomassen M, Tan Q, Bak M, Cold S, Burton M, et al. Long non-coding RNA HOTAIR is an independent prognostic marker of metastasis in estrogen receptor-positive primary breast cancer. Breast cancer research and treatment. 2013;142(3):529-36.

61. Chisholm KM, Wan Y, Li R, Montgomery KD, Chang HY, West RB. Detection of long non-coding RNA in archival tissue: correlation with polycomb protein expression in primary and metastatic breast carcinoma. PloS one. 2012;7(10):e47998.

62. Xue X, Yang YA, Zhang A, Fong K, Kim J, Song B, et al. LncRNA HOTAIR enhances ER signaling and confers tamoxifen resistance in breast cancer. Oncogene. 2016;35(21):2746-55.

63. Zhao W, Geng D, Li S, Chen Z, Sun M. Lnc RNA HOTAIR influences cell growth, migration, invasion, and apoptosis via the miR‐20a‐5p/HMGA 2 axis in breast cancer. Cancer medicine. 2018;7(3):842-55.

64. Ren Y, Jia H-h, Xu Y-q, Zhou X, Zhao X-h, Wang Y-f, et al. Paracrine and epigenetic control of CAF-induced metastasis: the role of HOTAIR stimulated by TGF-ss1 secretion. Molecular cancer. 2018;17(1):1-14.

65. Ding W, Ren J, Ren H, Wang D. Long noncoding RNA HOTAIR modulates MiR-206-mediated Bcl-w signaling to facilitate cell proliferation in breast cancer. Scientific reports. 2017;7(1):1-9.

66. Wang Y, Gong G, Xu J, Zhang Y, Wu S, Wang S. Long noncoding RNA HOTAIR promotes breast cancer development by targeting ZEB1 via sponging miR-601. Cancer Cell International. 2020;20(1):1-13.

67. Lozano-Romero A, Astudillo-de la Vega H, Terrones-Gurrola MCdR, Marchat LA, Hernández-Sotelo D, Salinas-Vera YM, et al. HOX transcript antisense RNA HOTAIR abrogates vasculogenic mimicry by targeting the AngiomiR-204/FAK axis in triple negative breast cancer cells. Non-coding RNA. 2020;6(2):19.

68. Wu D, Zhu J, Fu Y, Li C, Wu B. LncRNA HOTAIR promotes breast cancer progression through regulating the miR-129-5p/FZD7 axis. Cancer Biomarkers. 2021;30(2):203-12.

69. Zhang S, Wang B, Xiao H, Dong J, Li Y, Zhu C, et al. LncRNA HOTAIR enhances breast cancer radioresistance through facilitating HSPA1A expression via sequestering miR‐449b‐5p. Thoracic cancer. 2020;11(7):1801-16.

70. Liu Y, Li M, Yu H, Piao H. lncRNA CYTOR promotes tamoxifen resistance in breast cancer cells via sponging miR‑125a‑5p. International journal of molecular medicine. 2020;45(2):497-509.

71. Zhang H, Zhao B, Wang X, Zhang F, Yu W. LINC00511 knockdown enhances paclitaxel cytotoxicity in breast cancer via regulating miR-29c/CDK6 axis. Life sciences. 2019;228:135-44.

72. Fan J, Xing Y, Wen X, Jia R, Ni H, He J, et al. Long non-coding RNA ROR decoys gene-specific histone methylation to promote tumorigenesis. Genome biology. 2015;16(1):1-17.

73. Hu A, Hong F, Li D, Jin Y, Kon L, Xu Z, et al. Long non-coding RNA ROR recruits histone transmethylase MLL1 to up-regulate TIMP3 expression and promote breast cancer progression. Journal of Translational Medicine. 2021;19(1):1-13.

74. Eades G, Wolfson B, Zhang Y, Li Q, Yao Y, Zhou Q. lincRNA-RoR and miR-145 Regulate Invasion in Triple-Negative Breast Cancer via Targeting ARF6lincRNA-RoR and miR-145 Regulate Invasion in Triple-Negative Breast Cancer. Molecular Cancer Research. 2015;13(2):330-8.

75. Hou L, Tu J, Cheng F, Yang H, Yu F, Wang M, et al. Long noncoding RNA ROR promotes breast cancer by regulating the TGF-β pathway. Cancer cell international. 2018;18(1):1-8.

76. Li Y, Jiang B, Zhu H, Qu X, Zhao L, Tan Y, et al. Inhibition of long non-coding RNA ROR reverses resistance to Tamoxifen by inducing autophagy in breast cancer. Tumor Biology. 2017;39(6):1010428317705790.

77. Peng W-x, Huang J-g, Yang L, Gong A-h, Mo Y-Y. Linc-RoR promotes MAPK/ERK signaling and confers estrogen-independent growth of breast cancer. Molecular cancer. 2017;16(1):1-11.

78. Zhang H-Y, Liang F, Zhang J-W, Wang F, Wang L, Kang X-G. Effects of long noncoding RNA-ROR on tamoxifen resistance of breast cancer cells by regulating microRNA-205. Cancer chemotherapy and pharmacology. 2017;79(2):327-37.

79. Liang Y, Li Y, Song X, Zhang N, Sang Y, Zhang H, et al. Long noncoding RNA LINP1 acts as an oncogene and promotes chemoresistance in breast cancer. Cancer biology & therapy. 2018;19(2):120-31.

80. Liu X, Yang B, Han J. Increased long noncoding RNA LINP1 expression and its prognostic significance in human breast cancer. Eur Rev Med Pharmacol Sci. 2018;22(24):8749-54.

81. Du S, Hu W, Zhao Y, Zhou H, Wen W, Xu M, et al. Long non-coding RNA MAGI2-AS3 inhibits breast cancer cell migration and invasion via sponging microRNA-374a. Cancer Biomarkers. 2019;24(3):269-77.

82. Xu S, Sui S, Zhang J, Bai N, Shi Q, Zhang G, et al. Downregulation of long noncoding RNA MALAT1 induces epithelial-to-mesenchymal transition via the PI3K-AKT pathway in breast cancer. International journal of clinical and experimental pathology. 2015;8(5):4881.

83. Kim J, Piao H-L, Kim B-J, Yao F, Han Z, Wang Y, et al. Long noncoding RNA MALAT1 suppresses breast cancer metastasis. Nature genetics. 2018;50(12):1705-15.

84. Chou J, Wang B, Zheng T, Li X, Zheng L, Hu J, et al. MALAT1 induced migration and invasion of human breast cancer cells by competitively binding miR-1 with cdc42. Biochemical and biophysical research communications. 2016;472(1):262-9.

85. Huang XJ, Xia Y, He GF, Zheng LL, Cai YP, Yin Y, et al. MALAT1 promotes angiogenesis of breast cancer. Oncology reports. 2018;40(5):2683-9.

86. Jin C, Lu Q, Lin Y, Ma L. Reciprocal regulation of Hsa-miR-1 and long noncoding RNA MALAT1 promotes triple-negative breast cancer development. Tumor Biology. 2016;37(6):7383-94.

87. Zuo Y, Li Y, Zhou Z, Ma M, Fu K. Long non-coding RNA MALAT1 promotes proliferation and invasion via targeting miR-129-5p in triple-negative breast cancer. Biomedicine & Pharmacotherapy. 2017;95:922-8.

88. Liu R, Li J, Lai Y, Liao Y, Liu R, Qiu W. Hsa-miR-1 suppresses breast cancer development by down-regulating K-ras and long non-coding RNA MALAT1. International journal of biological macromolecules. 2015;81:491-7.

89. Miao Y, Fan R, Chen L, Qian H. Clinical significance of long non-coding RNA MALAT1 expression in tissue and serum of breast cancer. Annals of Clinical & Laboratory Science. 2016;46(4):418-24.

90. Wang Y, Zhou Y, Yang Z, Chen B, Huang W, Liu Y, et al. MiR-204/ZEB2 axis functions as key mediator for MALAT1-induced epithelial–mesenchymal transition in breast cancer. Tumor Biology. 2017;39(7):1010428317690998.

91. Zhang P, Zhou H, Lu K, Lu Y, Wang Y, Feng T. Exosome-mediated delivery of MALAT1 induces cell proliferation in breast cancer. OncoTargets and therapy. 2018;11:291.

92. Li Z, Xu L, Liu Y, Fu S, Tu J, Hu Y, et al. LncRNA MALAT1 promotes relapse of breast cancer patients with postoperative fever. American journal of translational research. 2018;10(10):3186.

93. Li J, Li Q, Li D, Shen Z, Zhang K, Bi Z, et al. Long non-coding RNA MNX1-AS1 promotes progression of triple negative breast cancer by enhancing phosphorylation of Stat3. Frontiers in oncology. 2020;10:1108.

94. Zhang W, Shi S, Jiang J, Li X, Lu H, Ren F. LncRNA MEG3 inhibits cell epithelial-mesenchymal transition by sponging miR-421 targeting E-cadherin in breast cancer. Biomedicine & Pharmacotherapy. 2017;91:312-9.

95. Zhang C-y, Yu M-s, Li X, Zhang Z, Han C-r, Yan B. Overexpression of long non-coding RNA MEG3 suppresses breast cancer cell proliferation, invasion, and angiogenesis through AKT pathway. Tumor Biology. 2017;39(6):1010428317701311.

96. Zhang J, Guo S, Jia B. Down-regulation of long non-coding RNA MEG3 serves as an unfavorable risk factor for survival of patients with breast cancer. Eur Rev Med Pharmacol Sci. 2016;20(24):5143-7.

97. Shi W, Xia S, Yin Y, Qi X, Xing C. Decreased expression of lncRNA MEG3 in breast cancer is associated with poor prognosis. Int J Clin Exp Pathol. 2016;9:5327-33.

98. Ouyang Q, Cui Y, Yang S, Wei W, Zhang M, Zeng J, et al. lncRNA MT1JP Suppresses Biological Activities of Breast Cancer Cells in vitro and in vivo by Regulating the miRNA-214/RUNX3 Axis. OncoTargets and therapy. 2020;13:5033.

99. Wu H, Li S. Long non-coding RNA MT1JP exerts anti-cancer effects in breast cancer cells by regulating miR-92-3p. General Physiology and Biophysics. 2020;39(1):59-67.

100. Li W, Zhang Z, Liu X, Cheng X, Zhang Y, Han X, et al. The FOXN3-NEAT1-SIN3A repressor complex promotes progression of hormonally responsive breast cancer. The Journal of clinical investigation. 2017;127(9):3421-40.

101. Li X, Deng S, Pang X, Song Y, Luo S, Jin L, et al. LncRNA NEAT1 silenced miR-133b promotes migration and invasion of breast cancer cells. International journal of molecular sciences. 2019;20(15):3616.

102. Li X, Wang S, Li Z, Long X, Guo Z, Zhang G, et al. The lncRNA NEAT1 facilitates cell growth and invasion via the miR-211/HMGA2 axis in breast cancer. International journal of biological macromolecules. 2017;105:346-53.

103. Zhao D, Zhang Y, Wang N, Yu N. NEAT1 negatively regulates miR-218 expression and promotes breast cancer progression. Cancer Biomarkers. 2017;20(3):247-54.

104. Shin VY, Chen J, Cheuk IW-Y, Siu M-T, Ho C-W, Wang X, et al. Long non-coding RNA NEAT1 confers oncogenic role in triple-negative breast cancer through modulating chemoresistance and cancer stemness. Cell death & disease. 2019;10(4):1-10.

105. Jiang X, Zhou Y, Sun AJ, Xue JL. NEAT1 contributes to breast cancer progression through modulating miR‐448 and ZEB1. Journal of cellular physiology. 2018;233(11):8558-66.

106. Liu B, Sun L, Liu Q, Gong C, Yao Y, Lv X, et al. A cytoplasmic NF-κB interacting long noncoding RNA blocks IκB phosphorylation and suppresses breast cancer metastasis. Cancer cell. 2015;27(3):370-81.

107. Shi X, Tang X, Su L. Overexpression of long noncoding RNA PTENP1 inhibits cell proliferation and migration via suppression of miR-19b in breast cancer cells. Oncology Research Featuring Preclinical and Clinical Cancer Therapeutics. 2018;26(6):869-78.

108. Chen S, Wang Y, Zhang J-H, Xia Q-J, Sun Q, Li Z-K, et al. Long non-coding RNA PTENP1 inhibits proliferation and migration of breast cancer cells via AKT and MAPK signaling pathways. Oncology letters. 2017;14(4):4659-62.

109. Yndestad S, Austreid E, Skaftnesmo KO, Lønning PE, Eikesdal HP. Divergent Activity of the Pseudogene PTENP1 in ER-Positive and Negative Breast CancerPTENP1 in Breast Cancer Progression. Molecular Cancer Research. 2018;16(1):78-89.

110. Mohebi M, Sattari A, Ghafouri-Fard S, Modarressi MH, Kholghi-Oskooei V, Taheri M. Expression profiling revealed up-regulation of three lncRNAs in breast cancer samples. Experimental and molecular pathology. 2020;117:104544.

111. Wang X, Li S, Xiao H, Deng X. Serum lncRNA TINCR serve as a novel biomarker for predicting the prognosis in triple-negative breast cancer. Technology in Cancer Research & Treatment. 2020;19:1533033820965574.

112. Li G-Y, Wang W, Sun J-Y, Xin B, Zhang X, Wang T, et al. Long non-coding RNAs AC026904. 1 and UCA1: a “one-two punch” for TGF-β-induced SNAI2 activation and epithelial-mesenchymal transition in breast cancer. Theranostics. 2018;8(10):2846.

113. Xiao C, Wu C, Hu H. LncRNA UCA1 promotes epithelial-mesenchymal transition (EMT) of breast cancer cells via enhancing Wnt/beta-catenin signaling pathway. Eur Rev Med Pharmacol Sci. 2016;20(13):2819-24.

114. Li Y, Zeng Q, Qiu J, Pang T, Xian J, Zhang X. Long non-coding RNA UCA1 promotes breast cancer by upregulating PTP1B expression via inhibiting miR-206. Cancer cell international. 2019;19(1):1-12.

115. Xu C, Yang M, Ren Y, Wu C, Wang L. Exosomes mediated transfer of lncRNA UCA1 results in increased tamoxifen resistance in breast cancer cells. Eur Rev Med Pharmacol Sci. 2016;20(20):4362-8.

116. Wu C, Luo J. Long non-coding RNA (lncRNA) urothelial carcinoma-associated 1 (UCA1) enhances tamoxifen resistance in breast cancer cells via inhibiting mTOR signaling pathway. Medical science monitor: international medical journal of experimental and clinical research. 2016;22:3860.

117. Li Z, Yu D, Li H, Lv Y, Li S. Long non‑coding RNA UCA1 confers tamoxifen resistance in breast cancer endocrinotherapy through regulation of the EZH2/p21 axis and the PI3K/AKT signaling pathway. International journal of oncology. 2019;54(3):1033-42.

118. Zhu H-y, Bai W-d, Ye X-m, Yang A-g, Jia L-t. Long non-coding RNA UCA1 desensitizes breast cancer cells to trastuzumab by impeding miR-18a repression of Yes-associated protein 1. Biochemical and biophysical research communications. 2018;496(4):1308-13.

119. Wang Y-H, Wang F, Zhang L, Lu J-C. Long non-coding RNA UCA1 can predict tumor lymph node metastasis. Tumor Biology. 2017;39(5):1010428317706208.

120. Zhu J, Kong F, Xing L, Jin Z, Li Z. Prognostic and clinicopathological value of long noncoding RNA XIST in cancer. Clinica chimica acta. 2018;479:43-7.

121. Xing F, Liu Y, Wu S-Y, Wu K, Sharma S, Mo Y-Y, et al. Loss of XIST in Breast Cancer Activates MSN-c-Met and Reprograms Microglia via Exosomal miRNA to Promote Brain MetastasisLoss of XIST Promotes Brain Metastasis. Cancer research. 2018;78(15):4316-30.

122. Huang Y-S, Chang C-C, Lee S-S, Jou Y-S, Shih H-M. Xist reduction in breast cancer upregulates AKT phosphorylation via HDAC3-mediated repression of PHLPP1 expression. Oncotarget. 2016;7(28):43256.

123. Yao J, Xu F, Zhang D, Yi W, Chen X, Chen G, et al. TP73‐AS1 promotes breast cancer cell proliferation through miR‐200a‐mediated TFAM inhibition. Journal of cellular biochemistry. 2018;119(1):680-90.

124. Ma Y, Bu D, Long J, Chai W, Dong J. LncRNA DSCAM‐AS1 acts as a sponge of miR‐137 to enhance Tamoxifen resistance in breast cancer. Journal of cellular physiology. 2019;234(3):2880-94.

125. Li Z-h, Yu N-s, Deng Q, Zhang Y, Hu Y-y, Liu G, et al. Lncrna snhg7 mediates the chemoresistance and stemness of breast cancer by sponging mir-34a. Frontiers in oncology. 2020;10:592757.
